# Active learning for prediction of tensile properties for material extrusion additive manufacturing

**DOI:** 10.1038/s41598-023-38527-6

**Published:** 2023-07-15

**Authors:** Tahamina Nasrin, Masoumeh Pourali, Farhad Pourkamali-Anaraki, Amy M. Peterson

**Affiliations:** 1 Department of Plastics Engineering, University of Massachusetts Lowell, Lowell, MA USA; 2 Department of Mathematical and Statistical Sciences, University of Colorado Denver, Denver, CO USA

**Keywords:** Mechanical engineering, Soft materials, Computer science

## Abstract

Machine learning techniques were used to predict tensile properties of material extrusion-based additively manufactured parts made with Technomelt PA 6910, a hot melt adhesive. An adaptive data generation technique, specifically an active learning process based on the Gaussian process regression algorithm, was employed to enable prediction with limited training data. After three rounds of data collection, machine learning models based on linear regression, ridge regression, Gaussian process regression, and K-nearest neighbors were tasked with predicting properties for the test dataset, which consisted of parts fabricated with five processing parameters chosen using a random number generator. Overall, linear regression and ridge regression successfully predicted output parameters, with < 10% error for 56% of predictions. K-nearest neighbors performed worse than linear regression and ridge regression, with < 10% error for 32% of predictions and 10–20% error for 60% of predictions. While Gaussian process regression performed with the lowest accuracy (< 10% error for 32% of prediction cases and 10–20% error for 40% of predictions), it benefited most from the adaptive data generation technique. This work demonstrates that machine learning models using adaptive data generation techniques can efficiently predict properties of additively manufactured structures with limited training data.

## Introduction

Additive manufacturing (AM) is a processing technique in which objects are fabricated in a layer-by-layer fashion. Fused filament fabrication (FFF) is the most common material extrusion based (MatEx) AM process, in which polymer filament is heated until molten, extruded through a nozzle, and deposited on a build surface to form three-dimensional parts. A detailed understanding of the FFF process, from the printability of the starting materials to the process-structure-properties relationships of the printed parts is required to control the final part quality. However, final part quality depends on many process parameters, complicating efforts towards quality control.

Physics-based models can provide important insights in understanding MatEx. These models are based on the relevant physics while setting assumptions and boundary conditions to closely reflect the original process. The broad ranges of models can be divided into distinct categories. Understanding heat transfer and thermal distribution during printing is important since they are critical to interlayer adhesion in printed parts. Finite volume^1^, finite element analysis (FEA)^2,3^, finite difference^3^, and numerical^4^ models of heat transfer in MatEx have been reported. Melt rheology has also been modeled, both within the hot end and of the extrudate^5–7^. Models have also been created for determination of final mechanical properties^8,9^. Physics-based models do not require large experimental datasets and may have high accuracy because they account for the relevant physics. However, they are often limited by their computational complexity, which is not only time-consuming but also requires comprehensive knowledge of multi-scale and multi-physics behaviors of the AM process^10^. As a result, models are restricted to only a few aspects of the entire process.

In data-driven machine learning (ML) models, a machine or system extracts underlying patterns from existing observations or experimental data to make predictions regarding new observations without the need for explicit programming. ML models can be less computationally intensive than physics-based models when the desired output is prediction of properties based on a multi-dimensional design space, such as the one typical to MatEx. Several review articles provide comprehensive overviews of the current state of ML applications in MatEx^10–12^. Artificial neural networks (ANNs) are the most prevalent method for AM process optimization^10^. ANN is more computationally efficient than FEA at investigating the role of infill parameters on mechanical properties such as tensile strength^13^ and strength to weight ratio^14^. Several studies focused on predicting macroscale mechanical properties such as tensile strength^15^, dynamic modulus of elasticity^16^, compressive stress^17^, and creep characteristics^18^ utilizing ANN-based multi-layer perceptron (MLP) models. ML models for optimizing dimensional accuracy^19^ and surface roughness^20^ based on input process parameters were created with an ANN-based model and an ensemble model, respectively.

Data-driven numerical solutions using ML techniques are more computationally efficient than physics-based numerical simulation. However, obtaining a sufficiently large dataset, which is a prerequisite for training high-quality ML models, can be expensive and time-consuming. Simple design of experiment (DOE) approaches uniformly sample from the entire design space, which exponentially increases experimental costs with increasing numbers of inputs^21^. Although the statement is strictly true only for simple DOE methods, slicing software (3D model-to-print software) packages for FFF include > 100 adjustable parameters, rendering non-iterative traditional DOE approaches for optimizing print parameters infeasible in many cases^22^. Moreover, DOEs such as Taguchi fall short because orthogonal arrays do not consider all variable combinations, which may omit important conditions from the model’s training^23^. Consequently, adaptive sampling is preferred over DOEs in cases like MatEx, where labeling outputs for each input datapoint is expensive. Adaptive sampling utilizing Bayesian optimization (BO), a form of active learning (AL), has attracted the attention of the material science community for reducing experimental/simulation effort while maximizing ML model accuracy by balancing tradeoffs between exploitation and exploration^24^. BO has been implemented extensively in material design/discovery and performance prediction for novel materials^25–29^. It has also been employed to generate adaptive experimental designs, which enabled the rapid and inexpensive exploration of datasets compared to DOEs^30^. However, implementation of BO in AM has only been reported recently. Process optimization through adaptive sampling has been reported for material development^31^, improving bond quality^32^, increasing geometry accuracy^33^, and optimizing mechanical properties such as surface roughness^34^ and toughness^35^ of MatEx parts.

In this work, we investigate an adaptive data generation process to obtain a training dataset for predicting properties of specimens printed using FFF. The material used is Technomelt PA 6910, a semicrystalline hot melt adhesive that we have previously shown is capable of forming void-free printed structures with properties that are comparable to, or even exceed, molded specimens^36^. Technomelt PA 6910 offers very different mechanical properties compared to the materials typically used in MatEx. This material is elastomeric, so property prediction requires determination of Young’s modulus, yield, and failure, which adds to complexity.

To the authors’ knowledge, this is the first instance of an uncertainty-based adaptive sampling scheme for generating an experimentally-acquired training dataset for simultaneously predicting five critical tensile properties rather than a single output for FFF parts. This is an important contribution since the proposed framework is an end-to-end ML pipeline starting with an efficient way to generate training data and ending with model evaluation. This framework, which can predict multiple quantities simultaneously, was used for prediction of tensile properties of FFF parts. Additionally, we provide a comprehensive evaluation of the trained ML models using testing samples collected independently from the training set. Unlike the most common approach of dividing the available data into train and test sets, we randomly selected the testing samples after completing the AL process. We also include a systematic analysis of printed structures including their void content analysis, crystallinity by differential scanning calorimetry (DSC), and cross-sectional microstructure through scanning electron microscopy (SEM). This type of model evaluation to consider sources of variations in AM problems is unique because most existing works resort to solely reporting standard evaluation metrics such as root mean squared error, which may not be as informative as a standalone metric. Hence, this paper describes principled approaches for capturing the complexity associated with the mechanical properties of elastomeric materials and predicting their tensile properties with low error utilizing regression models trained with tensile data from just 22 printing conditions. This work thus provides grounding for future work in the area of data-driven modeling in AM.

## Materials and methods

### Materials

A polyamide-based hot melt adhesive, Technomelt PA 6910, was used in this study. Technomelt PA 6910 is a semicrystalline flexible thermoplastic with a sub-ambient glass transition temperature and melt temperature above 60 °C^36^. It is designed to reach bond strength rapidly upon cooling, which is advantageous for hot melt adhesive applications as well as in material extrusion AM. Henkel Corporation kindly provided the material in the form of strand cut pellets.

### Filament extrusion

Filament was extruded using a Dr. Collin single screw extruder (COLLIN Lab & Pilot Solutions GmbH). A 3.5 mm diameter die was used to extrude filaments having a diameter of 2.85 ± 0.06 mm. Details of the extrusion process parameters are available in previous work^36^.

### Fused filament fabrication (FFF)

ASTM D638-14 type-V tensile bars were printed with an Ultimaker 3 FFF printer at an environmental temperature of 22 ± 1 °C. The extruder nozzle has a diameter of 0.4 mm. G-code was generated in Cura 4.3. Samples were oriented along the XY plane, meaning the largest surface area of specimens was in contact with the print bed. Print bed temperature and infill were set to 60 °C and 110%, respectively, for all prints. All samples were printed at 30 mm/s because good quality prints were obtained at this print speed. Raster angle, the angle between the direction of infill roads relative to the X-axis of the print bed, was randomly selected to be 45° or 90°. In previous work, the yield stress (σ_y_), yield strain (ε_y_), and ultimate tensile stress (σ_f_) of FFF-printed Technomelt PA 6910 were shown to be independent of raster angle. Ultimate tensile strain (ε_f_) was shown to be statistically significantly lower for parts with 0° raster angle, although all results are within a small range of (1104–1265%)^36^. We subsequently evaluated the tensile data to determine the variance in Young's modulus with the raster angles and observed that it was statistically insignificant (Supporting Information, Fig. S1). A JEOL JSM 6390 (JEOL USA Inc.) was used to collect SEM images of cryogenically fractured cross-sections of the FFF tensile bars.

### Differential scanning calorimetry (DSC)

DSC was performed on printed specimens using a Discovery DSC (TA Instruments). Samples were taken from the gauge length of the printed tensile bars and conditions were tested in triplicate. Samples were prepared in aluminum hermetic pans and were then heated from − 50 to 250 °C, cooled to − 90 °C and again heated to 250 °C at heating and cooling rates of 10 K/min.

### Density measurement

Density measurements were performed in triplicate of filament and tensile samples from each test condition. These measurements were performed using a density determination kit (Sartorius AG) with ethanol as the liquid medium. The ethanol temperature was measured for determining its density $(\rho (f)$). For each sample/filament, weight in air ($W(a)$) and weight in ethanol ($W(f)$**)** were measured. Density ($\rho$) was calculated according to Eq. (1).

1 $$\uprho =\frac{\mathrm{W}\left(\mathrm{a}\right).\uprho (\mathrm{f})}{\mathrm{W}\left(\mathrm{a}\right)-\mathrm{W}(\mathrm{f})}$$

### Dataset generation

The training dataset used in this work is available in Table S1 and the print conditions for the training samples are summarized in Table S2. The initial training samples, acquired from previous study by Pourali and Peterson^36^, are labeled as iteration 1. For all subsequent iterations, extruder temperature (T_ext_) ranged from 200 to 240 °C and layer height (h) ranged from 0.06 to 0.4 mm. 25 possible combinations were generated by dividing the T_ext_ and h ranges into five equally spaced values. This approach enforced reasonable spacing between the training data points. In addition, finer discretization would increase the likelihood that the entire design space would not be explored due to experimental cost. A GPR-based AL process aided in identifying the additional training sample print conditions, which are labeled as iteration 2 and iteration 3. The GPR algorithm was used to identify the five parameter conditions with the highest level of uncertainty, which were then taken as the print conditions for the next iterations. S1.2 provides a theoretical explanation of AL with GPR.

Since the design space was reduced to only 25 points, the top five conditions were chosen. If the design space was divided into finer regions, selecting the top five conditions with the highest level of uncertainty would not be adequate to explore all the areas, as the training data collection procedure for this work is extremely time-consuming. To provide a sense of the time required to experimentally acquire one data point for the training dataset, it takes approximately 25 min to print one tensile bar using the FFF process with selected printing parameters, in addition to another 10 min to perform tensile testing on it and analyze the results. Thus, selecting only the five printing conditions with the highest level of uncertainty for iterations 2 and 3 drastically reduced the amount of time required to acquire a viable training dataset.

**Figure 1:**
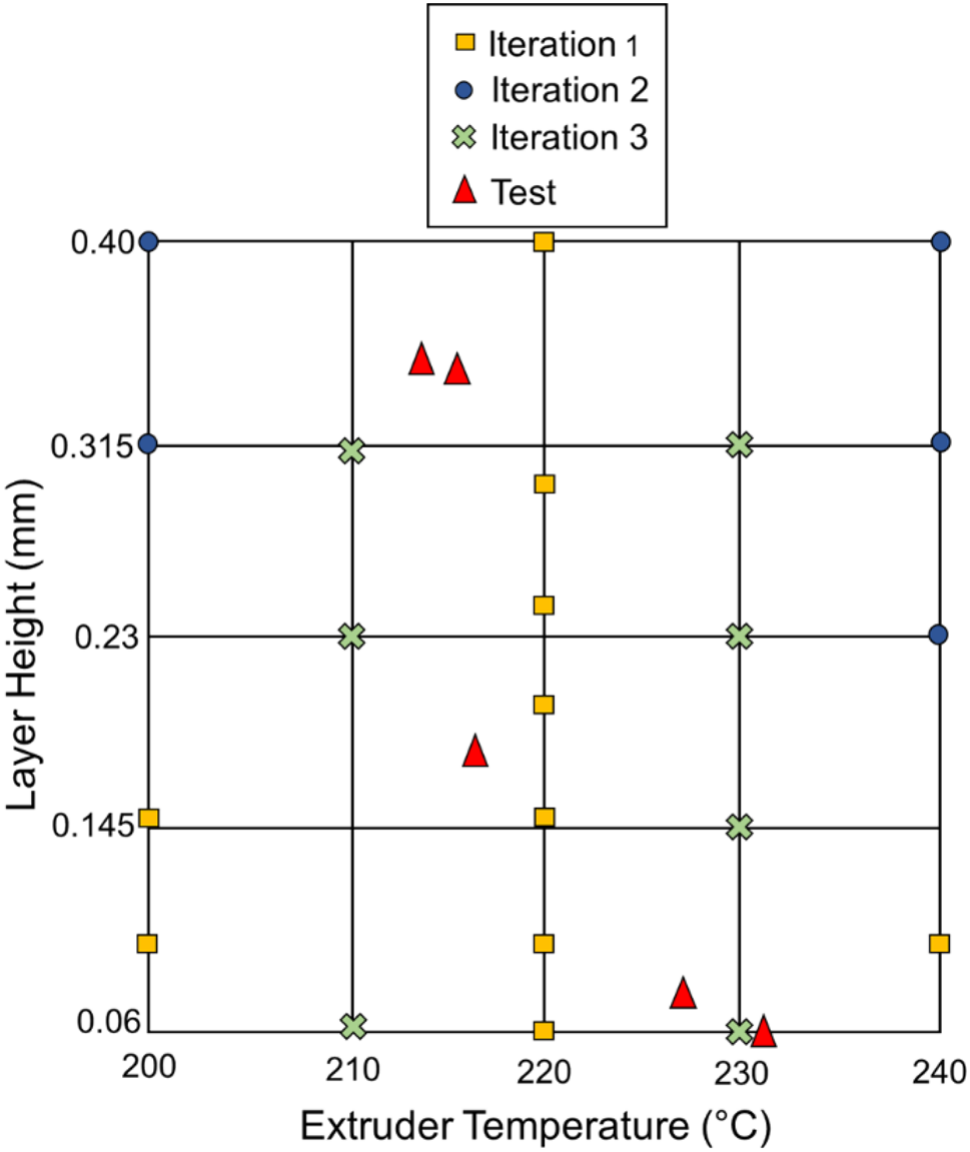
Print conditions for iterations 1, 2, and 3 and testing samples.

A random number generator was used to determine test sample print conditions to assess the performance of trained ML models. Our objective was to evaluate the performance of the model using test data that is truly distinct from the training data. T_ext_ and h were limited to the same ranges as the training data (200–240 °C and 0.06–0.4 mm, respectively) but not from the 25 conditions generated by discretization of the design space. The random sampling method resulted in print conditions that were less uniformly distributed than if an orthogonal method such as Latin hypercube sampling (LHS) was used. We used random sampling in order to produce a small number of test samples for determining the accuracy of the predictive models. It was necessary to restrict the number of required test cases because the data were derived from physical experiments that required considerable time and effort to generate samples, perform mechanical testing, and analyze the results. Table 1 shows the print parameter conditions for the test samples. Six specimens were printed per condition. Figure 1 illustrates print conditions used for training ML models and testing samples oriented throughout the parameter space for evaluation purposes.

**Table 1:** Print parameter conditions for testing data to evaluate ML models.

Print condition	T (°C)	h (mm)	θ (°)
A	231	0.06	45
B	217	0.18	45
C	214	0.36	45
D	216	0.34	90
E	226	0.08	90

### Tensile testing

Tensile testing was conducted on an Instron 4481 with a 10 kN load cell in accordance with ASTM D638-14. The crosshead speed was set to 75 mm/min so that the rupture times fell within the recommended time range of 30 s to 5 min. 2% offset was considered while determining the yield point.

### Input and output parameters for ML models

**Figure 2:**
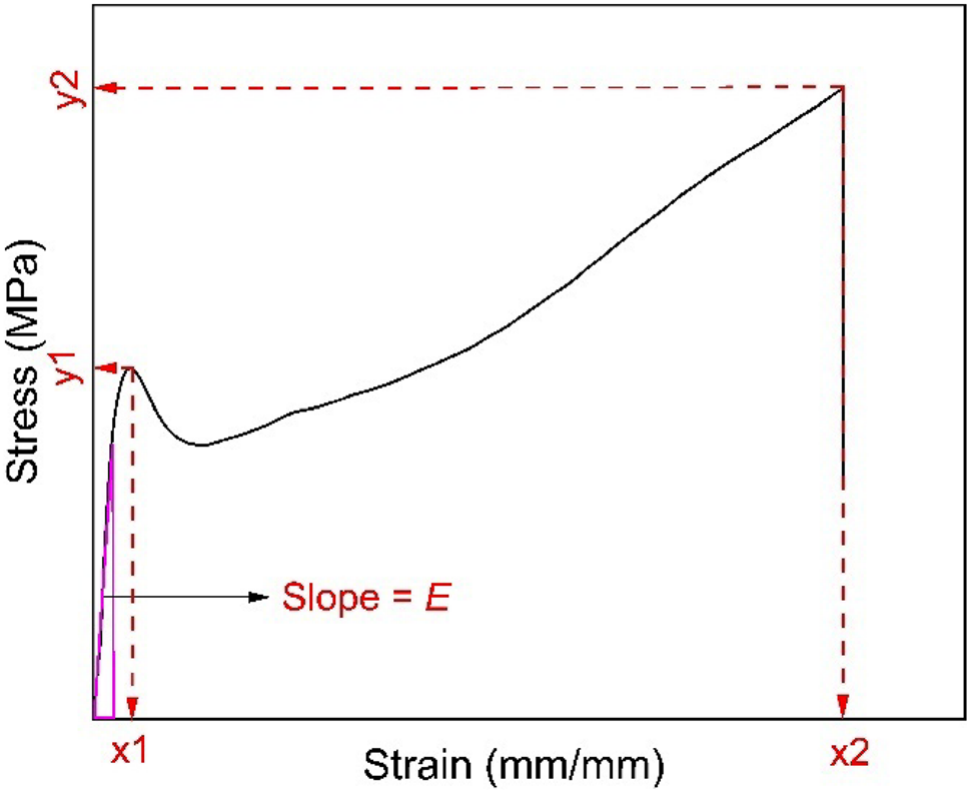
Typical stress–strain curve with model output parameters highlighted.

The input parameters were T_ext_ and h. The output parameters, which are shown in Fig. 2, were Stress 1 (y1), Strain 1 (x1), Stress 2 (y2), Strain 2 (x2), and Young’s modulus ($E$). y1 and  x1 are slightly higher than σ_y_ and ε_y_, respectively, while y2 and x2 are equivalent to σ_f_ and ε_f_, respectively. These output parameters were selected because (1) they represent or approximate important tensile properties; (2) combined, these parameters represent the key features of a stress–strain curve; and (3) they can be easily and reproducibly determined from experimental tensile data, which aids in data processing for training data and assessment of model accuracy for testing data.

### Machine learning (ML) approach

The aim of the study was to evaluate the performance of regression models for the prediction of $E$, y1, y2, x1, and x2. A total of 124 samples from iterations 1, 2, and 3 were taken as training data for four regression techniques, namely linear regression (LR), ridge regression (RR), Gaussian process regression (GPR), and K-nearest neighbors (KNN). LR was used as the baseline model to compare the prediction quality of the regression models. All regression models were taken from scikit-learn, which is an open-source machine learning library for the Python programming language^37^. MinMaxScalar from scikit-learn was used as a preprocessing step to scale the features to be between zero and one^38^. The RR algorithm in this work used degree-3 polynomial features. A radial-basis (RBF) kernel function was used in the GPR algorithm to model similarities between data samples. For the KNN regression algorithm, the number of nearest neighbors was taken to be 10. These regression approaches are described in greater detail in S1.1.

For the AL strategy used in this work, we began with 47 training data points, which were categorized as iteration 1. 26 of these data points were previously reported by Pourali and Peterson^36^. GPR was used to estimate the uncertainties associated with iteration 1 to give the print parameters for iteration 2 samples, which were the samples associated with conditions of highest output uncertainty. The results from iteration 2 were input to the model, and GPR was used to estimate the new model uncertainty and identify the processing parameters associated with the highest uncertainty—these conditions were then chosen for iteration 3. Combined, these data were used to train the ML models that then predicted the discussed output parameters for the test data conditions and the accuracy of the models was assessed by comparing the model results to the experimental results. A diagram of this process is given in Fig. S2.

## Results and discussion

### Training dataset

**Figure 3:**
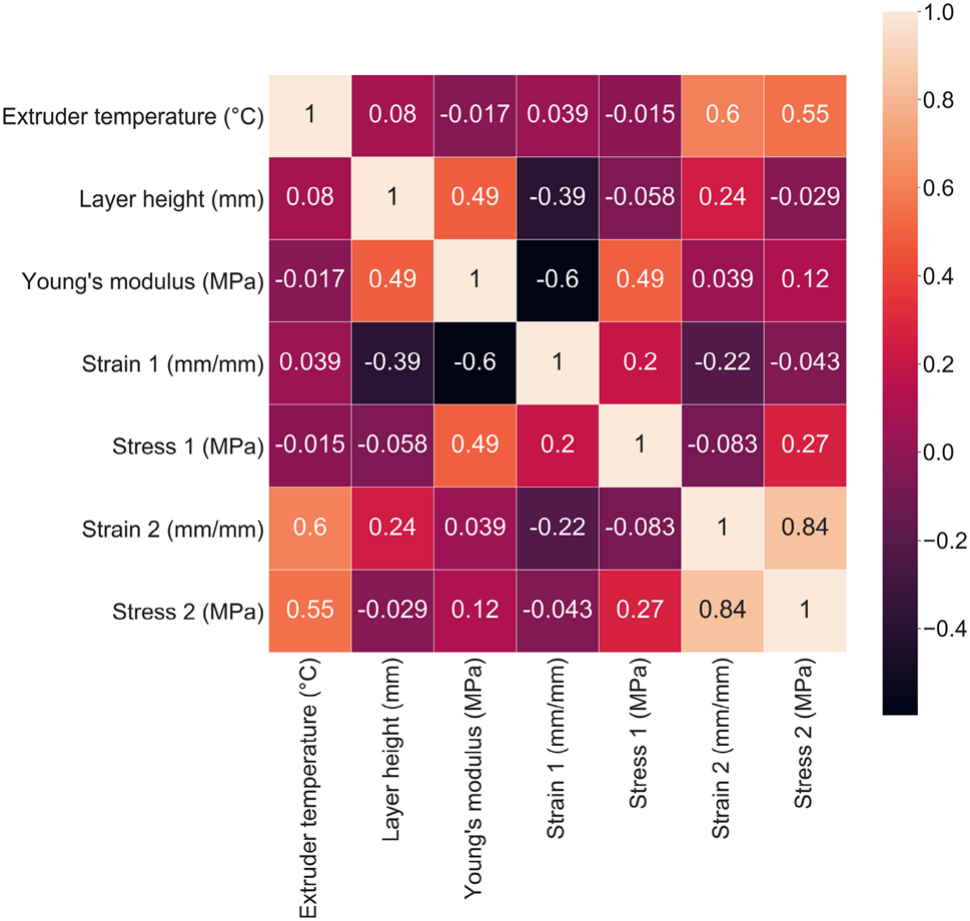
Heatmap showing correlations between the input and output parameters of the training dataset.

The training dataset, which was taken from Pourali and Peterson as well as experiments that led to the determination of print parameters to use in that study, was first investigated for correlations between print parameters (inputs) and mechanical properties (outputs) and for correlations between different mechanical properties^36^. A heatmap, shown in Fig. 3, was generated using the algorithm available in the seaborn library.

T_ext_ is positively correlated with y2 and x2, which are equivalent to σ_f_ and ε_f_, respectively. This result is consistent with previous work in which ultimate stress and strain increased with higher T_ext_ due to higher interlayer adhesion^36^. h is positively correlated with $E$ but negatively correlated with x1. In this work, h ranged from 0.06 to 0.4 mm. Previous work shows that the mechanical properties of Technomelt PA 6910 improve as h increases from 0.06 mm to 0.15 mm due to increased contact between the adjacent roads. T_ext_ affected mechanical properties more than h, which was also observed by Pourali and Peterson.

Considering the output parameters, $E$ is positively correlated with y1 but negatively correlated with x1. x2 and y2 are strongly positively correlated. Based on these correlations between the different output variables, $E$ and y2 could be used to estimate the other output variables of a given sample printed at a specific print condition.

### Test sample tensile properties

The test conditions can be divided into two categories: (1) Higher T_ext_/lower h (conditions A and E) and (2) Lower T_ext_/higher h (conditions B, C, and D). Figure S3 shows stress–strain curves from tensile testing of specimens printed at the test conditions. Mechanical properties determined based on these data are summarized in Table 2, along with void content and DSC results. ε_y_ occurred at approximately 30% strain for all conditions.

**Table 2:** Summary of tensile properties, void content, and thermal properties of test samples.

Print condition	E (MPa)	σ_y_ (MPa)	ε_y_ (%)	σ_f_ (MPa)	ε_f_ (%)	Voids (%)	ΔH_m_ (J/g)	T_m_ (°C)
A	38.3 ± 1.6	10.4 ± 0.5	32.5 ± 1.6	21.2 ± 0.9	939 ± 41	1.39 ± 0.03	21.8 ± 0.3	83.2 ± 1.2
B	40.6 ± 5.1	10.9 ± 0.6	32.1 ± 1.5	18.6 ± 3.3	810 ± 167	2.07 ± 0.11	20.4 ± 1.4	82.4 ± 0.3
C	39.2 ± 1.0	11.2 ± 0.7	31.8 ± 2.3	16.1 ± 2.1	809 ± 93	3.37 ± 0.19	18.9 ± 0.6	82.9 ± 0.5
D	40.5 ± 1.5	11.2 ± 0.6	31.1 ± 0.8	21.0 ± 1.9	1030 ± 95	3.65 ± 0.08	22.7 ± 0.7	83.1 ± 0.5
E	47.2 ± 1.3	13.2 ± 0.2	33.4 ± 0.7	25.2 ± 1.6	1089 ± 68	1.35 ± 0.10	22.4 ± 0.3	81.9 ± 0.3

Values represent mean ± standard deviation.

**Figure 4:**
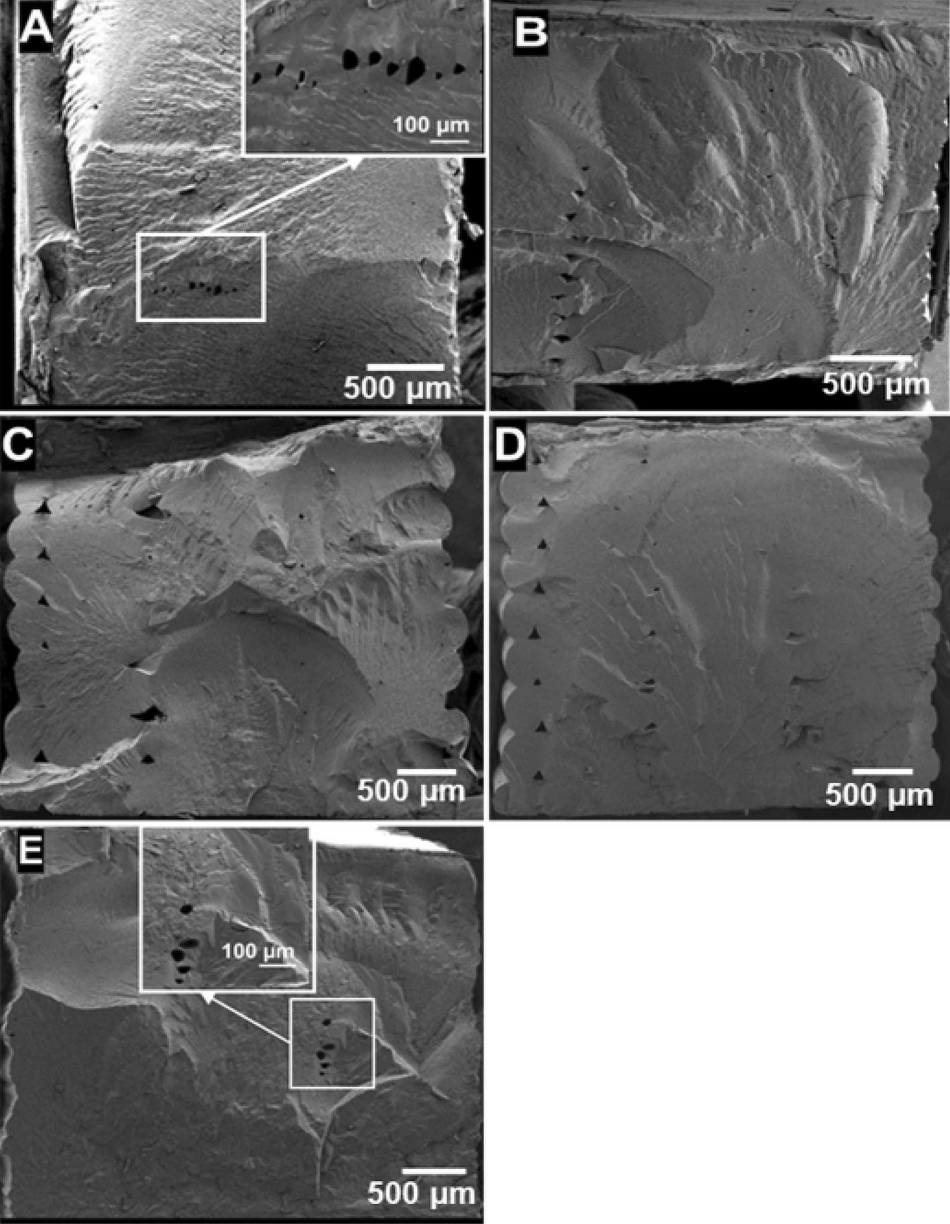
SEM images of cryogenically fractured surfaces of test samples printed at conditions A, B, C, D, and E.

Condition E samples had the highest $E$ and σ_f_, which indicates excellent adhesion between the layers. Although condition A samples have print parameters (T_ext_ and h) that are very close to those of condition E, they have statistically significantly lower $E$ (38.3 ± 1.6 vs. 47.2 ± 1.3 MPa) and failed at statistically significantly lower σ_f_ (21.2 ± 0.9 vs. 25.2 ± 1.6%) at a 95% confidence interval. This indicates that intralayer adhesion was worse in condition A samples^39^. Category 1 (A and E) samples had the lowest void percentages. Figure 4 shows SEM images of cryogenically fractured samples. Conditions A and E resulted in well-consolidated samples with no apparent inter/intralayer voids between the deposited roads. This is due, in part, to the low material viscosity at high extruder temperatures. However, some smaller voids were observed that may be attributed to volatiles that evaporated during printing. Although SEM does not show volatile-induced voids for condition B, C, and D samples, it is important to note that similar voids may be present in those samples, as SEM only provides information about a single cross-section. The reason behind the lower tensile properties of condition A samples compared to condition E samples could not be determined from their densities or fracture surfaces.

Category 2 (conditions B, C, and D) samples had higher void percentages than category 1 (condition A and E) samples. Higher void content can lead to worse mechanical properties in FFF^40^. Condition B and C samples’ higher void contents are consistent with their lower tensile strengths. The fracture surfaces of condition B and C samples in Fig. 4 also show many voids between roads. However, condition D exhibited comparable tensile strength to condition A, which had the lowest void fraction. The nature and location of the voids in condition D samples were different from condition B and C samples, with voids in condition D samples appearing closer to print walls, leaving large areas in the center void-free. These voids do not affect the tensile strength as much as those in condition B and C samples.

Table 2 also shows the melting enthalpy and melting temperatures (T_m_) of the samples printed at the test conditions. Representative heating curves are provided in Fig. S5a. T_m_ values are in the same range for all samples. However, melting enthalpies differ to some extent. Condition C samples have the lowest melting enthalpy, which could be a cause of condition C’s low tensile strength. Interestingly, condition D samples had significantly higher melting enthalpy compared to condition C samples even though the T_ext_ and h values are in the same range. Condition C samples were re-printed at a raster angle of 90° and their melting enthalpy was found to be 20.5 ± 0.3 J/g, which is much closer to other print conditions (Fig. S5b) and confirms that the difference in crystallinity is due to raster angle. Technomelt PA 6910 is capable of slowly crystallizing at room temperature, so crystallinity values should reach similar values over hours at room temperature, assuming they start with the same number of nucleation sites. These results suggest that certain combinations of T_ext_, h, and toolpath choices for a specific geometry may lead to flow-induced crystallization in Technomelt PA 6910. We also tried to determine the reason for the apparent higher tensile properties of condition E samples compared to condition A samples using DSC analysis. There was no statistically significant difference between the melting enthalpies of the samples from these two conditions, so differences between conditions A and E cannot be attributed to differences in crystallinity. The choice of raster angle may affect the tensile properties of samples from conditions A and E. Condition A, B, C, and D samples exhibited statistically indistinguishable $E$. $E$ of condition E samples was higher than the others. The T_ext_ values for condition B, C, and D samples were very close to each other, which likely explains their similar ε_y_, σ_y_, and $E$. Condition E had higher T_ext_ than condition B, C, and D. This could result in higher interlayer adhesion, giving higher $E$. Further increases in T_ext_ resulted in lower $E$, which was observed for condition A samples.

Figure S4 shows the correlations between the input and output parameters in the test dataset. Similar correlations between T_ext_ and the outputs were observed in both training and test datasets. However, the correlations between h and the outputs in the test dataset were not in complete agreement with the training dataset. Specifically, no correlation was observed between h and y2 in the training dataset, while a pronounced negative correlation was observed between them in the test dataset. The correlations between the output parameters were similar in both datasets. Overall, these correlations indicate that the training and test datasets behave similarly.

### ML model prediction and evaluation

**Figure 5:**
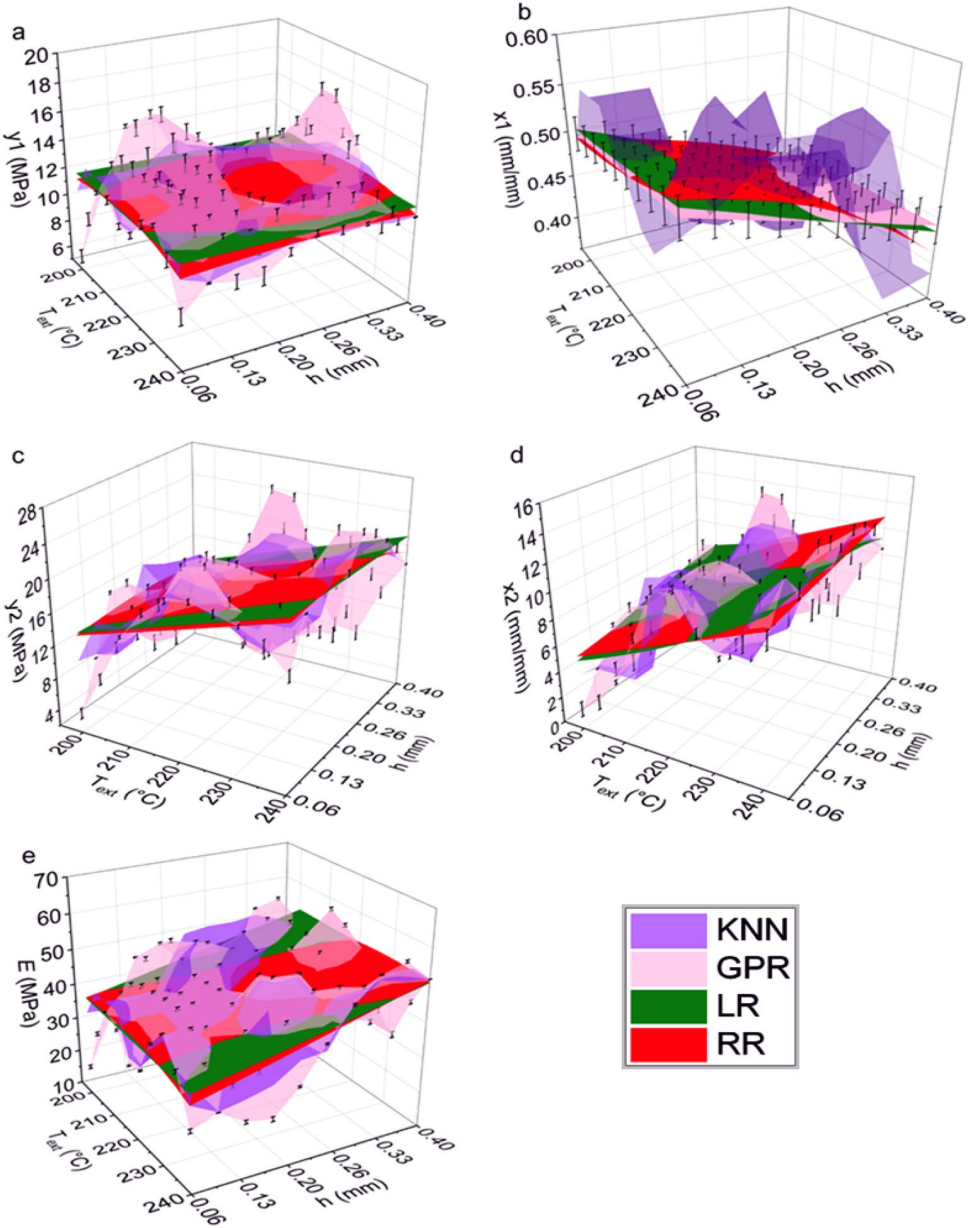
Surface plots for showing the relationship between inputs (T_ext_ and h) and various predicted outputs [(**a**) y1; (**b**) x1; (**c**) y2; (**d**) x2; and (**e**) *E*] as generated by different regression models (KNN, GPR, RR, and LR) within the input parameter ranges defined in Fig. 1. For GPR, error bars represent the prediction uncertainty.

**Figure 6:**
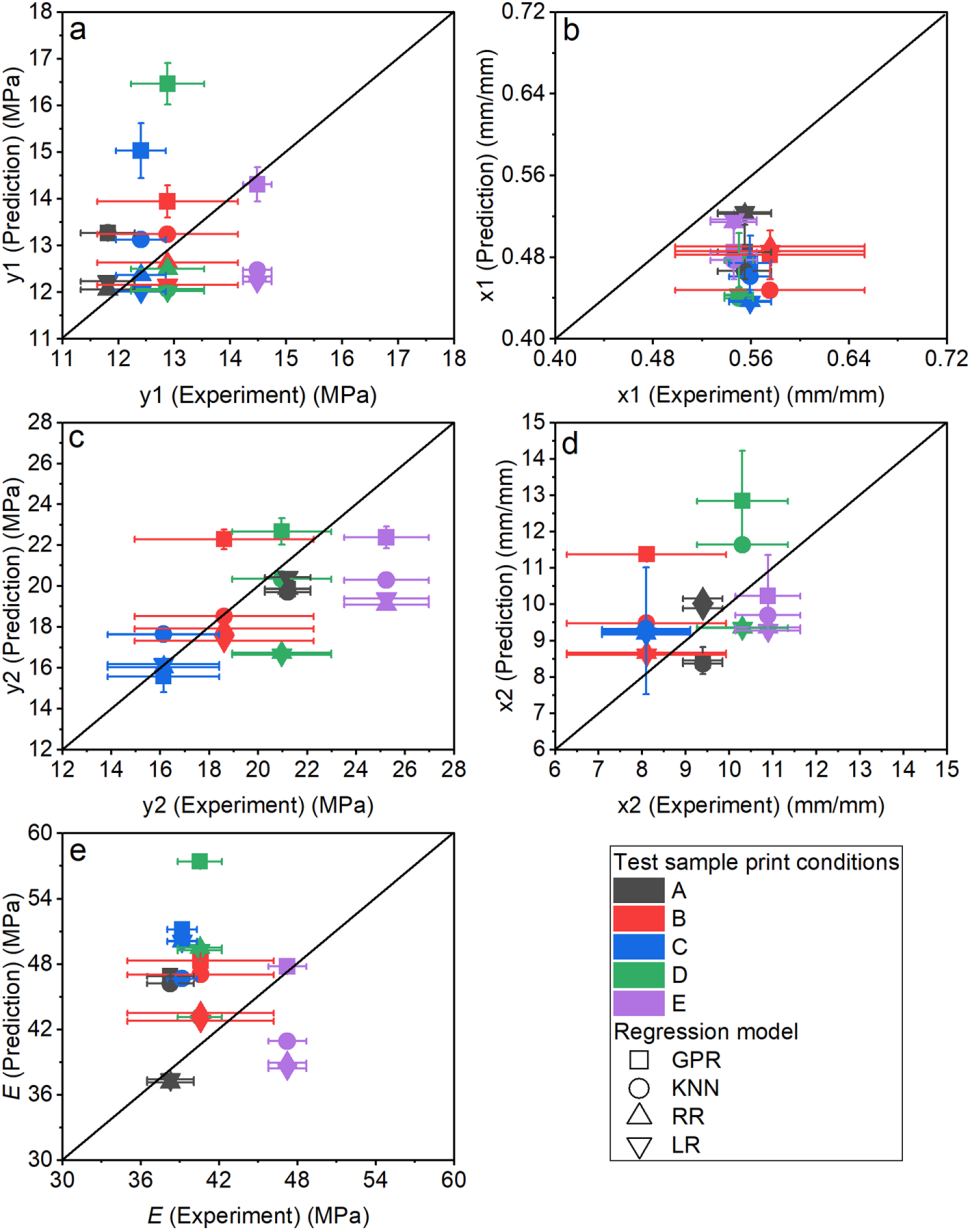
Assessment of model accuracy by comparing the experimental and predicted output values [(**a**) y1; (**b**) × 1; (**c**) y2; (**d**) × 2; and (**e**) *E*] from GPR, KNN, RR, and LR regression models for the samples printed at test conditions (A, B, C, D, and E).

The functional relationships between predicted output variables (y1, x1, y2, x2, and $E$) and input variables (T_ext_ and h) are depicted in Fig. 5. The LR surfaces for all the predicted outputs were observed to be completely flat. Using a three-degree polynomial for RR resulted in non-linearity, consistent with the curved surface plot. This observation is consistent with the well-known bias-variance tradeoff in ML, in which a model with fewer degrees of freedom typically suffers from higher bias (i.e., missing the relevant input–output patterns). KNN and GPR yielded surfaces with local maxima and minima. Predicted outputs at the test conditions were compared against the experimental values to evaluate prediction quality of the regression models in Fig. 6. The data in Fig. 6 is also available as bar charts in Fig. S6. For the subsequent discussion of model prediction quality for different outputs, we consider 0 to 10% error as good performance, 11 to 20% as moderate performance, and 21 to 40% as poor performance. These ranges were chosen since they encompass the full range of errors observed.

#### Prediction of y1

LR and RR models predicted that y1 is independent of T_ext_ and h. GPR predicted that there will be local maxima and minima in y1. GPR predicted the lowest y1 at the lowest T_ext_ (200 °C) and lowest h (0.06 mm) process parameter combination, whereas the greatest y1 values were predicted at T_ext_ = 227–222 °C and h = 0.36 mm. GPR also predicted that, at high T_ext_ (235–240 °C), changing h has no effect on y1. KNN predicted y1 to be within the range of 10–15 MPa, regardless of the processing parameter settings, with a small amount local variation in y1.

LR and RR showed good predictive performance for conditions A, B, C, and D and moderate performance for condition E. LR and RR predicted y1 for all the testing conditions to be 12–12.6 MPa, independent of T_ext_ or h. The experimental y1 values for conditions A, B, C, and D were consistent with these predictions, falling between 12 and 12.8 MPa with no statistically significant differences at a 95% confidence interval. Condition E samples had higher y1 than other print conditions, which explains why LR and RR underpredicted y1 for condition E. GPR and KNN performed worse than LR and RR at predicting y1. GPR’s predicted range for y1 overlapped with the experimental ranges for y1 for test conditions B and E; moderate performance was observed for condition A; and poor performance was observed for conditions C and D. GPR outperformed all other models at predicting y1 for condition E. KNN showed good performance at predicting y1 for conditions B, C, and D and moderate performance for conditions A and E. KNN overpredicted y1 for condition A and underpredicted for condition E. A possible reason is that KNN weighs the nearest neighbors in the training dataset more than the distant points. Test conditions A and E are very close to training condition 19 (T_ext_ = 230 °C and h = 0.06 mm), which had a mean y1 of 13.3 MPa. KNN predicted values close to 13.3 MPa for test conditions A and E, which had y1 values of 12.5 MPa and 13.3 MPa, respectively.

#### Prediction of x1

LR, RR, and GPR predicted that x1 is not a function of T_ext_. These models also predicted that the lowest value of h will result in the highest x1. KNN predicted x1 would fall between 0.4 and 0.6 mm/mm. KNN predicted the maximum x1 would occur at T_ext_ = 226–235 °C and h = 0.14–0.17 mm, while the lowest x1 values were predicted for T_ext_ = 240 °C with h = 0.32–0.4 mm. KNN also predicted that a high value of x1 (0.06 mm/mm) could be achieved when h ranged from 0.06 to 0.1 mm at lower values of T_ext_ (203–213 °C).

LR and RR exhibited good predictive performance for conditions A and E; moderate performance for conditions B and D; and poor performance for condition C. As shown in Fig. 5b, LR and RR predicted higher x1 with increased T_ext_, and consequently predicted higher x1 for the higher T_ext_ samples (conditions A and E) and lower x1 for lower T_ext_ samples (conditions B, C, and D). LR and RR underpredicted y1 for conditions B, C, and D. GPR performed moderately at predicting x1 under all test conditions. Experimental x1 values were consistent for all test samples, with an average value of 55.7 ± 1.02% strain. Only GPR successfully captured this behavior, as it was the only model that predicted the x1 values in the same range for all conditions, even though they were underpredicted by around 13%. KNN performed moderately for all test conditions except for condition B prints, for which it showed poor performance. The best performance of KNN was for condition E, which is very close to training condition 19 (T_ext_ of 230 °C and h of 0.06 mm), with an average x1 of 45%. KNN predicted 47.7% for condition E’s x1, which represents a 13% underprediction from the experimental x1 value of 54.6%.

#### Prediction of y2

Both LR and RR predicted that T_ext_ influences y2, but h does not. GPR exhibited peaks and troughs in the surface plot for y2 and predicted the lowest y2 for the lowest T_ext_ (200 °C) and the lowest h (0.06 mm), while the maximum y2 was predicted at T_ext_ = 217–222 °C and h = 0.32 mm. Similar to LR and RR, KNN predicted a general positive correlation with T_ext_.

LR and RR showed good performance at predicting y2 for conditions A, B, and C and poor performance for condition E. For condition D, RR showed moderate predictive performance, while LR showed poor performance. From the heatmap in Fig. 5, y2 and T_ext_ were found to be strongly positively correlated. This could be the reason LR and RR predicted higher y2 for higher T_ext_ print conditions and captured the experimental values for condition A, B, and C. KNN is the best performing model for predicting y2. Good performance was observed for conditions A, B, C, and D and moderate performance was observed for condition E. KNN accurately predicted y2 for condition B and predicted y2 for condition D with only 3% error. Test condition B is close to training conditions 5 (T_ext_ = 220 °C and h = 0.20 mm) and 6 (T_ext_ = 220 °C and h = 0.15 mm). y2 for test condition B samples is very close to the average for training conditions 5 and 6, which is 19.18 MPa—the similarity to conditions that are in proximity explain why KNN performed impressively at predicting condition B’s y2 with 0.5% error. Similarly, KNN’s prediction for condition D’s y2 (20.3 MPa) is close to y2 for training condition 7 (T_ext_ = 220 °C and h = 0.30 mm), which is 21 MPa. GPR outperformed LR and RR, with good predictive performance for conditions A, C, and D and moderate performance for conditions B and E. All models underpredicted y2 for test Condition E, although GPR performed the best with 11% error.

In experiments, the highest tensile strength (y2) was observed for condition E samples and the lowest was observed for condition C samples. The low y2 for condition C is likely due to it having the lowest T_ext_. The lowest T_ext_ combined with the highest h produced the highest amount of interlayer voids and lowest melt enthalpy/crystallinity. Tensile strength is positively correlated with T_ext_ across a wide range of polymer feedstocks because it increases interlayer welding^41–43^. h is the highest for condition C, which may be why a low T_ext_ played a more important role than h in dictating tensile strength. There is a lack of consensus in the literature regarding the effect of h on tensile strength, with both negative^43–45^ and positive^46–48^ correlations reported. We do not observe a correlation between h and y2 in the heatmap of the training dataset; however, a negative correlation was observed in the testing dataset (Fig. S4). This discrepancy highlights a limitation of using a small training dataset. The models failed to learn the correlations between h and y2 from the training dataset due to insufficient granularity. Hence, LR, RR and KNN underpredicted y2 for condition E. Both LR and RR predicted that condition A would have the highest y2, which seemed plausible since condition A had the highest T_ext_ among the test conditions. Instead, the highest y2 value was obtained from condition E, which RR failed to capture, most likely because the training dataset did not have raster orientation as one of the input features.

#### Prediction of ×2

LR and RR predicted, similar to y2, that T_ext_ has a greater influence on x2 than h. GPR predicted the lowest x2 values for the lowest T_ext_ (200 °C) and lowest h (0.06 mm) combination, and the greatest x2 values for T_ext_ = 217—222 °C and h = 0.32 mm. KNN predicted that the highest x2 would be obtained from T_ext_ = 235–240 °C and h ~ 0.1 mm. LR and RR showed good performance for predicting x2 for conditions A, B, and D and moderate performance for conditions C and E. The experimental x2 values for conditions A, D, E are significantly higher than conditions B and C. However, condition D is similar to condition C in terms of both T_ext_ and h. The higher experimental x2 values for conditions A and E compared to B and C are expected due to conditions A and E’s higher T_ext_. Higher T_ext_ improved interlayer diffusion and reduced voids between roads, which resulted in higher tensile properties and failure at higher ultimate strain/x2. LR and RR were somewhat successful at capturing this experimental trend, predicting higher x2 values for conditions A, D, and E compared to B and C. Although LR and RR predicted similar x2 for conditions D and E, the experimental x2 value for condition E was significantly higher than condition D. KNN performed moderately well under all print conditions. GPR showed good performance for conditions A and E; moderate for condition C prints; and poor for conditions B and D. GPR and KNN both predicted that x2 would be higher for condition D than condition E, which disagrees with experimental results.

#### Prediction of *E*

LR and RR both predicted that $E$ is more affected by h than T_ext_. GPR also predicted a somewhat positive trend of $E$ with h. Similar to y1, GPR predicted the highest $E$ would be obtained with T_ext_ in the range of 227–222 °C and h = 0.36 mm. In contrast to GPR, KNN predicted that $E$ would be comparatively lower in T_ext_ range of 217–222 °C. The highest $E$ (52–54 MPa) was observed for T_ext_ = 235–240 °C and h = 0.36–0.4 mm as well as T_ext_ = 226–231 °C and h = 0.2–0.25 mm. At relatively lower T_ext_ (200–213 °C), KNN predicted high values of $E$ (~ 50 MPa) for h = 0.2–0.4 mm.

The experimental $E$ values were very close (38–40 MPa) for conditions A, B, C, and D. We observed higher $E$ value for condition E. Even though conditions A and E are very similar, $E$ for condition E was substantially higher than condition A. This is surprising and cannot be explained by void content, void location, or melting enthalpy.

LR and RR showed good performance at predicting $E$ for conditions A and B; moderate for condition E; and poor for conditions C and D. KNN showed good performance for condition D only; moderate for conditions B, C, and E; and poor for condition A. GPR showed good performance for condition E only; moderate for condition B; and poor for conditions A, C, and D. No single model showed good prediction performance for all print conditions. $E$ for conditions A and B are best predicted by LR and RR. All the models overpredicted $E$ for condition C and predicted that this condition would have the highest $E$ among all the conditions. Only KNN showed moderate prediction performance, with 19% error for this condition.

From the heatmap in Fig. 4, there is a strong correlation between $E$ and h, but little to no correlation between $E$ and T_ext_. The surface plot in Fig. 5e shows that the models predicted $E$ independent of T_ext_. A positive correlation between T_ext_ and $E$ was expected in the training dataset, since increased T_ext_ should provide better fusion between the layers to result in higher tensile property samples^41–43^. The small training dataset could explain why a correlation between $E$ and T_ext_ was not observed in the data or models. However, it is also plausible that elastomeric systems behave differently than room temperature glassy materials such that T_ext_ does not affect *E*. This observation highlights the complexity of predicting mechanical properties of additively manufactured elastomeric materials as well as an opportunity for future investigation. LR and RR predicted the lowest $E$ values for condition A and E since they had the smallest h values. These conditions were also similar, so the predicted values were very close. LR and RR were successful at capturing $E$ for condition A but failed for condition E, since condition E samples exhibited higher than expected $E$ values. KNN captured $E$ of the condition D samples because this condition is very close to training condition 7 (T_ext_ = 220 °C and h = 0.3 mm). GPR overpredicted $E$ values for all test conditions, which is why it was successful at capturing conditions E’s much higher $E$.

### Iteration-dependent prediction quality of the regression models

**Figure 7:**
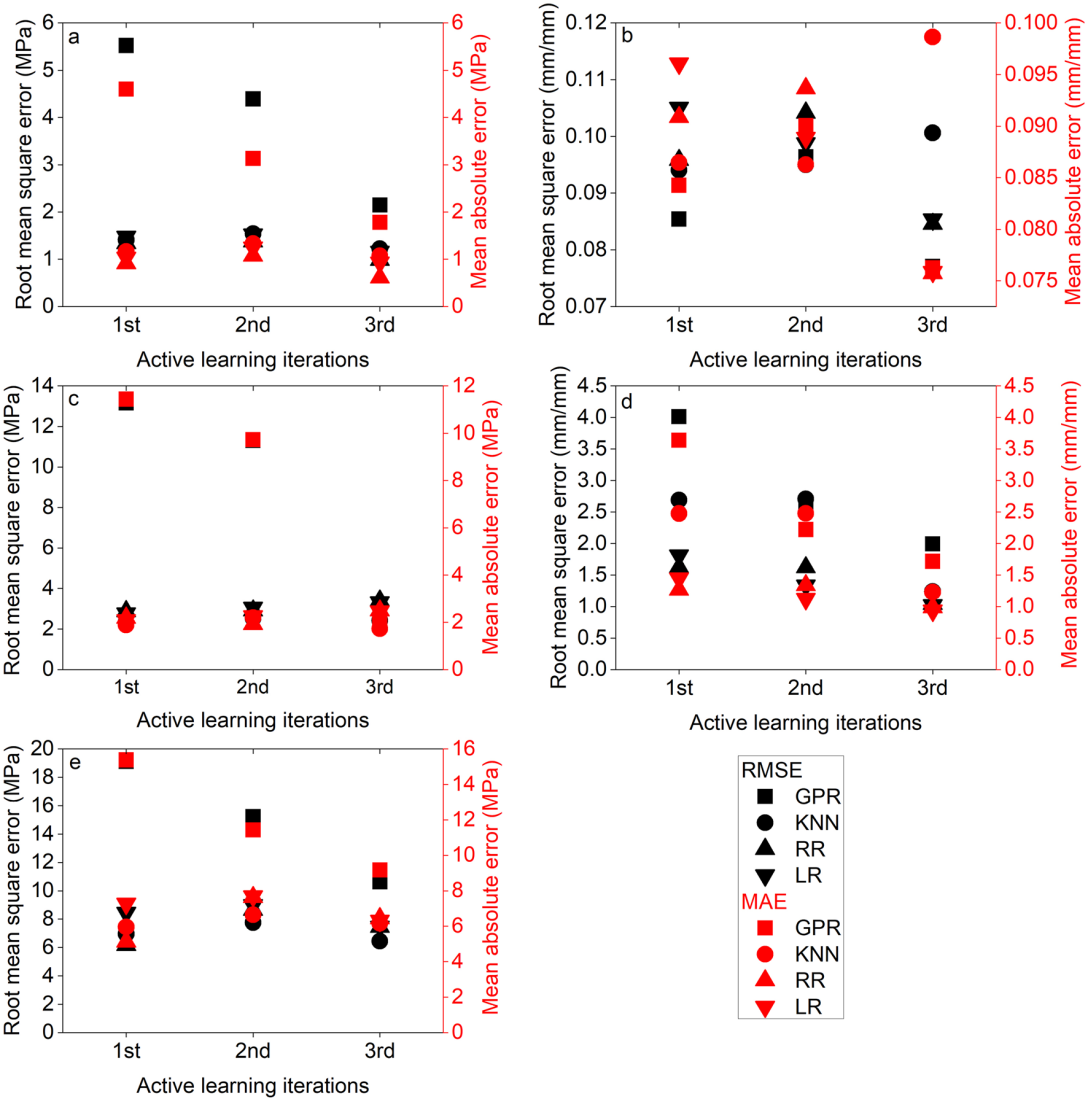
Root mean squared error (RMSE) and mean absolute error (MAE) values obtained across three iterations of active learning for predicting (**a**) y1; (**b**) x1; (**c**) y2; (**d**) x2; and (**e**) *E* with GPR, KNN, LR, and RR.

At each iteration of the adaptive data generation technique, root mean squared error (RMSE) and mean absolute error (MAE) were calculated to evaluate the prediction performance of the regression models and are shown in Fig. 7.

When trained with the dataset from the first iteration, LR, RR, and KNN performed similarly at predicting y1 based on comparable RMSE and MAE values, while GPR performed poorly. When trained with the second iteration dataset, the RMSE and MAE values for LR, RR, and KNN unexpectedly increased compared to the first iteration dataset. That may have been because five of iteration 2's print conditions were located on the perimeter of the design space, as opposed to the print conditions for the testing samples that were concentrated in the middle of the design space. In contrast, GPR considerably benefited from the adaptive generation method. When trained with the second iteration dataset, the RMSE and MAE values decreased by 20% and 32%, respectively, but remained greater than LR, RR, and KNN. Due to comparatively homogeneous sampling of the design space, the RMSE and MAE values of all models trained with the third iteration dataset decreased. GPR displayed the highest reduction in RMSE and MAE values (61%) when compared to LR, RR, and KNN, demonstrating that it benefits the most from this AL process. Equivalent RMSE and MAE values indicate that LR, RR, and KNN all performed comparably. Although GPR benefitted significantly from the AL process, its final RMSE and MAE values after the third iteration were still greater than those of the other models, indicating poorer performance. Additional adaptive sampling may aid GPR in enhancing its prediction quality, allowing it to outperform other regression models.

Comparable RMSE and MAE values demonstrate that all models had comparable performance in predicting x1 across the first, second, and third iterations. The quality of predictions was almost unaffected by the iterative training data collection approach. The first iteration's training samples were adequate for the model to learn and provide accurate predictions for x1 with minimum error.

All models but GPR were penalized after the first iteration when predicting y2. The RMSE and MAE values for y2 at the third iteration significantly increased for LR (by 20% and 18%, respectively) and RR (by 18% and 14%, respectively). LR, RR, and KNN learned best from the training dataset of the first iteration due to these datapoints being closer to the test datapoints than the datapoints from iteration 2 and 3. Similar to y1, GPR demonstrated poor performance when trained using the first iteration training dataset, with high RMSE and MAE values. RMSE and MAE decreased by 14% and 15%, respectively, in the second iteration. However, these values reduced significantly (both by 82%) and became comparable to those of LR, RR, and KNN when GPR was trained with data from third iteration.

When trained with data from the third iteration, all models, particularly GPR and KNN, showed significant improvement in predicting x2. At the second iteration, both RMSE and MAE for GPR decreased significantly (by 34% and 19%, respectively), and this trend continued for the third iteration. KNN did not demonstrate improved performance until it was trained using the third iteration dataset, most likely due to the third iteration’s datapoints that are in close proximity to test datapoints.

The best performance in predicting $E$ was observed for RR when trained on data from the initial iteration. LR benefitted considerably from the third iteration, whereas RR was penalized. GPR benefited considerably from the adaptive data generation technique and shown a constant drop in the RMSE and MAE values with each successive iteration, but exhibited inferior performance in comparison to LR, RR, and KNN.

## Conclusions

AL, an adaptive data generation technique, was used to generate a training dataset for FFF samples printed with Technomelt PA 6910 to train LR, RR, KNN, and GPR models. The adaptive data generation scheme used in this study enabled simultaneous prediction of five critical tensile properties of FFF structures with acceptable accuracy. Most tensile properties were predicted with less than 10% error. However, the regression models failed to predict some tensile properties of test samples with high-level accuracy due to the small training dataset used in the study, resulted from coarse discretization (5 × 5) of the design space. Consequently, the predictive models failed to capture the crystallinity or void-induced change in tensile characteristics within smaller ranges of printing parameters than the predefined granularity of the T_ext_ and h distributions. The base model (LR) provided accurate predictions for the majority of output parameters, demonstrating that simple linear models are adequate for predicting tensile properties when combined with adaptive data generation technique. RR performed comparably to LR and sometimes showed higher performance than LR. KNN performed moderately when compared to LR and RR, with the fewest instances of under- and over-prediction. GPR underperformed compared to the other regression models; however, GPR benefitted the most from the AL process, and more sampling through the iterative process could result in GPR outperforming the other models.

The non-uniformity associated with random sampling for test data generation presented an opportunity in this study to investigate similar print conditions, such as A and E or B and D, which resulted in varying tensile properties that were not adequately captured by most predictive models. Sampling orthogonally, on the other hand, may produce test conditions with printing parameters that are too far apart from each other, limiting the ability to observe the anomalous tensile behavior. DSC and SEM analysis of the test samples offered valuable insights regarding the change in crystallinity and voids in FFF parts printed with varying parameters. Analyzing the crystallinity and nature/location of voids in the test samples revealed how they influenced the tensile characteristics, even with slight alterations in T_ext_ and h. By quantifying crystallinity and void content and subsequently including such data into the training process and/or increasing the granularity of the design space to accommodate additional training data points, the prediction quality of the models could be enhanced. However, incorporating the information would increase the cost of collecting training data.

This work showed the efficacy of AL techniques for predicting important tensile properties of a highly complicated system such as FFF of an elastomeric material by generating a small but informative dataset with only tensile properties corresponding to 22 print combinations. AL significantly reduced the experimentation effort. This process could be useful to optimize printing parameters of an unknown material with much less experimental time and effort. In addition, the proposed method offers a straightforward strategy that can be easily applied to a variety of problems. Its primary strength is its ability to selectively identify and concentrate on process parameter combinations associated with the highest uncertainty in each iteration. This is particularly advantageous for AM techniques. Typically, AM processes involve numerous process parameters that significantly influence part properties. Understanding the intricate relationships between the process parameters and part properties can be challenging, specifically when multiple properties are of interest. Therefore, our method can be especially valuable in minimizing the number of experiments required for simultaneous optimization of process parameters for multiple properties.

The sampling strategy includes taking the average uncertainty across normalized outputs for aggregating the uncertainty information, which yielded reasonable accuracy for predicting all the studied tensile properties. In other words, the correlations between the outputs were disregarded in order to simplify the prediction problem, and it was assumed that outputs were predicted independently. As a direction for future research, the sampling strategy could be enhanced by appropriately incorporating the correlations in the output space. Future investigation may also include a systematic comparison of the performance of models trained with datasets generated by the proposed method with grid-based sampling techniques, such as LHS and stratified sampling, or random sampling techniques, such as Monte Carlo sampling. Another possible area of future research could be whether LHS combined with uniform sampling can provide more representative samples than a pure uniform sampling strategy. The outcome of this investigation will depend on various problem-specific characteristics, such as the number of input parameters, their ranges, and the total number of selected samples.

## Supplementary Information

Supplementary Information.

## Data Availability

Model inputs are provided in the supporting information. Additional data are available at 10.18126/Y4E2-BYV0.
