# Supplementary material for: Active learning for prediction of tensile properties for material extrusion additive manufacturing
Source: Sci Rep. 2023 Jul 15;13:11460. doi: 10.1038/s41598-023-38527-6 (PMC10349846; doi:10.1038/s41598-023-38527-6)
Supplement: Supplementary file 1 — Supplementary Information. [file 41598_2023_38527_MOESM1_ESM.docx]

**Supplementary Information for “Active learning for prediction of tensile properties for material extrusion additive manufacturing”**


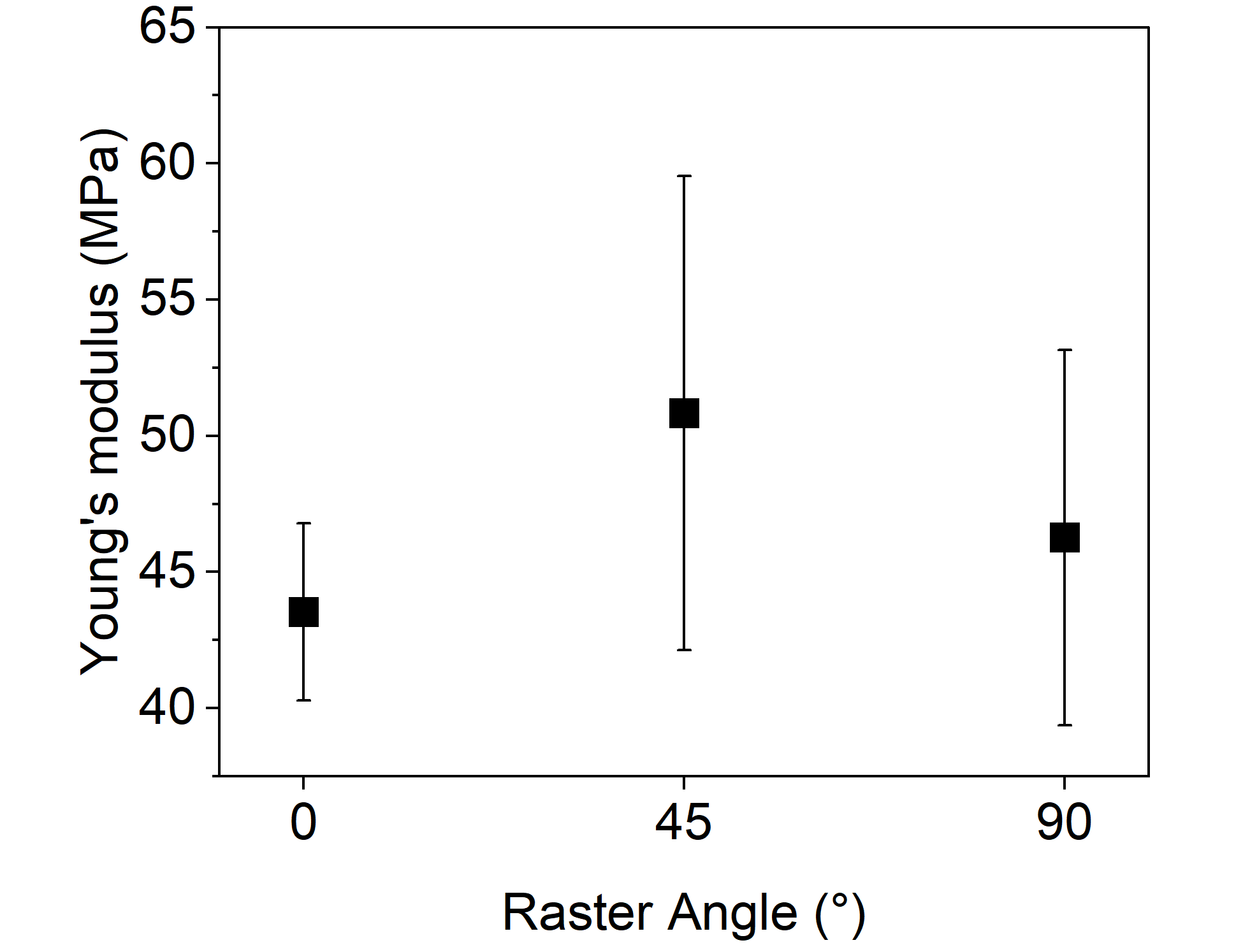


**Figure S1.** Young’s modulus of samples printed at T_ext_ of 220°C with different raster angles. Error bars represent 95% confidence interval.

**Table S1.** Training dataset

| Sample no | Iteration | Print speed (mm/s) | T_ext_ (°C) | Print bed temperature (°C) | h (mm) | Raster angle (°) | Infill (%) | $E$  (MPa) | x1 (mm/mm) | y1 (MPa) | x2 (mm/mm) | y2 (MPa) |
| --- | --- | --- | --- | --- | --- | --- | --- | --- | --- | --- | --- | --- |
| 1 | 1 | 30 | 220 | 60 | 0.15 | 45 | 110 | 51.0 | 0.4 | 14.6 | 11.7 | 25.1 |
| 2 | 1 | 30 | 220 | 60 | 0.15 | 45 | 110 | 49.3 | 0.4 | 13.3 | 6.5 | 13.2 |
| 3 | 1 | 30 | 220 | 60 | 0.15 | 45 | 110 | 41.9 | 0.5 | 12.1 | 6.0 | 12.0 |
| 4 | 1 | 30 | 220 | 60 | 0.15 | 45 | 110 | 40.8 | 0.4 | 11.0 | 9.8 | 16.2 |
| 5 | 1 | 30 | 220 | 60 | 0.25 | 45 | 110 | 25.6 | 0.4 | 7.0 | 7.6 | 7.1 |
| 6 | 1 | 30 | 220 | 60 | 0.1 | 45 | 110 | 50.7 | 0.5 | 14.5 | 11.9 | 24.4 |
| 7 | 1 | 30 | 220 | 60 | 0.2 | 45 | 110 | 49.1 | 0.5 | 14.1 | 12.2 | 23.9 |
| 8 | 1 | 30 | 220 | 60 | 0.3 | 45 | 110 | 52.4 | 0.5 | 14.7 | 12.3 | 24.8 |
| 9 | 1 | 30 | 220 | 60 | 0.4 | 45 | 110 | 52.7 | 0.5 | 14.8 | 7.2 | 13.3 |
| 10 | 1 | 30 | 200 | 60 | 0.15 | 45 | 110 | 50.8 | 0.5 | 15.8 | 7.1 | 18.0 |
| 11 | 1 | 30 | 220 | 60 | 0.15 | 45 | 110 | 49.8 | 0.5 | 14.7 | 11.1 | 24.7 |
| 12 | 1 | 30 | 220 | 60 | 0.15 | 45 | 110 | 48.5 | 0.4 | 12.5 | 8.9 | 19.1 |
| 13 | 1 | 30 | 220 | 60 | 0.15 | 45 | 110 | 44.4 | 0.4 | 12.2 | 11.4 | 21.7 |
| 14 | 1 | 30 | 220 | 60 | 0.15 | 45 | 110 | 45.8 | 0.5 | 14.1 | 11.7 | 23.3 |
| 15 | 1 | 30 | 220 | 60 | 0.15 | 45 | 110 | 42.7 | 0.5 | 12.5 | 10.8 | 20.5 |
| 16 | 1 | 30 | 220 | 60 | 0.15 | 45 | 110 | 44.4 | 0.4 | 12.7 | 11.5 | 22.3 |
| 17 | 1 | 30 | 220 | 60 | 0.15 | 45 | 110 | 46.6 | 0.4 | 13.4 | 8.4 | 15.9 |
| 18 | 1 | 30 | 220 | 60 | 0.15 | 90 | 110 | 50.0 | 0.5 | 13.5 | 8.4 | 15.8 |
| 19 | 1 | 30 | 220 | 60 | 0.15 | 45 | 110 | 47.1 | 0.4 | 12.8 | 8.5 | 14.9 |
| 20 | 1 | 30 | 200 | 60 | 0.1 | 90 | 110 | 29.7 | 0.5 | 9.8 | 5.0 | 10.0 |
| 21 | 1 | 30 | 200 | 60 | 0.1 | 90 | 110 | 37.6 | 0.5 | 10.7 | 2.2 | 9.0 |
| 22 | 1 | 30 | 200 | 60 | 0.1 | 90 | 110 | 45.5 | 0.4 | 11.9 | 2.8 | 10.2 |
| 23 | 1 | 30 | 200 | 60 | 0.1 | 90 | 110 | 23.9 | 0.6 | 10.2 | 5.1 | 10.9 |
| 24 | 1 | 30 | 200 | 60 | 0.1 | 90 | 110 | 26.4 | 0.5 | 11.0 | 3.1 | 9.6 |
| 25 | 1 | 30 | 220 | 60 | 0.3 | 90 | 110 | 37.2 | 0.5 | 10.5 | 13.4 | 20.5 |
| 26 | 1 | 30 | 220 | 60 | 0.3 | 90 | 110 | 37.7 | 0.4 | 11.2 | 13.1 | 21.7 |
| 27 | 1 | 30 | 220 | 60 | 0.3 | 90 | 110 | 41.5 | 0.5 | 10.9 | 13.1 | 20.7 |
| 28 | 1 | 30 | 220 | 60 | 0.3 | 90 | 110 | 42.3 | 0.4 | 11.1 | 12.7 | 21.1 |
| 29 | 1 | 30 | 220 | 60 | 0.3 | 90 | 110 | 42.4 | 0.4 | 11.4 | 13.0 | 21.4 |
| 30 | 1 | 30 | 220 | 60 | 0.3 | 90 | 110 | 38.1 | 0.4 | 11.0 | 13.0 | 21.4 |
| 31 | 1 | 30 | 220 | 60 | 0.06 | 90 | 110 | 26.2 | 0.5 | 10.4 | 13.1 | 22.4 |
| 32 | 1 | 30 | 220 | 60 | 0.06 | 90 | 110 | 20.5 | 0.6 | 11.0 | 12.9 | 21.7 |
| 33 | 1 | 30 | 220 | 60 | 0.06 | 90 | 110 | 23.8 | 0.6 | 11.2 | 13.5 | 22.5 |
| 34 | 1 | 30 | 220 | 60 | 0.06 | 90 | 110 | 34.6 | 0.4 | 10.6 | 12.4 | 21.2 |
| 35 | 1 | 30 | 220 | 60 | 0.06 | 90 | 110 | 33.7 | 0.5 | 11.1 | 10.4 | 17.7 |
| 36 | 1 | 30 | 220 | 60 | 0.06 | 90 | 110 | 35.6 | 0.5 | 11.0 | 13.3 | 21.7 |
| 37 | 1 | 30 | 240 | 60 | 0.1 | 90 | 110 | 35.7 | 0.5 | 11.2 | 13.2 | 20.9 |
| 38 | 1 | 30 | 240 | 60 | 0.1 | 90 | 110 | 32.0 | 0.4 | 10.0 | 14.3 | 22.4 |
| 39 | 1 | 30 | 240 | 60 | 0.1 | 90 | 110 | 45.2 | 0.4 | 11.3 | 13.6 | 21.2 |
| 40 | 1 | 30 | 240 | 60 | 0.1 | 90 | 110 | 21.0 | 0.6 | 10.5 | 12.4 | 19.0 |
| 41 | 1 | 30 | 240 | 60 | 0.1 | 90 | 110 | 18.7 | 0.7 | 10.3 | 14.2 | 22.1 |
| 42 | 1 | 30 | 240 | 60 | 0.1 | 90 | 110 | 28.6 | 0.5 | 9.9 | 14.5 | 22.4 |
| 43 | 1 | 30 | 200 | 60 | 0.1 | 90 | 110 | 38.5 | 0.4 | 10.7 | 2.9 | 8.8 |
| 44 | 1 | 30 | 200 | 60 | 0.1 | 90 | 110 | 49.8 | 0.4 | 12.2 | 4.2 | 10.7 |
| 45 | 1 | 30 | 200 | 60 | 0.1 | 90 | 110 | 19.4 | 0.6 | 10.5 | 5.6 | 12.4 |
| 46 | 1 | 30 | 200 | 60 | 0.1 | 90 | 110 | 32.6 | 0.5 | 11.4 | 5.5 | 12.6 |
| 47 | 2 | 30 | 240 | 60 | 0.23 | 90 | 110 | 35.2 | 0.6 | 9.9 | 12.0 | 22.3 |
| 48 | 2 | 30 | 240 | 60 | 0.23 | 90 | 110 | 32.0 | 0.7 | 11.0 | 11.7 | 23.8 |
| 49 | 2 | 30 | 240 | 60 | 0.23 | 90 | 110 | 34.3 | 0.6 | 10.9 | 12.7 | 24.9 |
| 50 | 2 | 30 | 240 | 60 | 0.23 | 90 | 110 | 37.7 | 0.6 | 12.5 | 13.3 | 27.7 |
| 51 | 2 | 30 | 240 | 60 | 0.23 | 90 | 110 | 38.8 | 0.6 | 13.1 | 11.6 | 24.4 |
| 52 | 2 | 30 | 240 | 60 | 0.23 | 90 | 110 | 39.5 | 0.5 | 12.6 | 11.8 | 25.2 |
| 53 | 2 | 30 | 200 | 60 | 0.315 | 45 | 110 | 52.6 | 0.4 | 11.1 | 6.8 | 12.0 |
| 54 | 2 | 30 | 200 | 60 | 0.315 | 45 | 110 | 50.2 | 0.4 | 11.0 | 6.4 | 10.9 |
| 55 | 2 | 30 | 200 | 60 | 0.315 | 45 | 110 | 55.1 | 0.4 | 11.8 | 10.1 | 17.2 |
| 56 | 2 | 30 | 200 | 60 | 0.315 | 45 | 110 | 54.5 | 0.4 | 11.4 | 8.3 | 13.7 |
| 57 | 2 | 30 | 200 | 60 | 0.315 | 45 | 110 | 52.3 | 0.4 | 10.8 | 7.5 | 12.4 |
| 58 | 2 | 30 | 200 | 60 | 0.315 | 45 | 110 | 56.0 | 0.4 | 11.4 | 6.6 | 11.1 |
| 59 | 2 | 30 | 240 | 60 | 0.315 | 45 | 110 | 56.1 | 0.4 | 11.6 | 12.9 | 22.1 |
| 60 | 2 | 30 | 240 | 60 | 0.315 | 45 | 110 | 53.1 | 0.4 | 11.2 | 14.1 | 23.2 |
| 61 | 2 | 30 | 240 | 60 | 0.315 | 45 | 110 | 54.9 | 0.4 | 11.2 | 13.6 | 23.2 |
| 62 | 2 | 30 | 240 | 60 | 0.315 | 45 | 110 | 55.7 | 0.4 | 12.9 | 13.3 | 23.7 |
| 63 | 2 | 30 | 240 | 60 | 0.315 | 45 | 110 | 52.6 | 0.4 | 11.2 | 13.0 | 22.4 |
| 64 | 2 | 30 | 240 | 60 | 0.315 | 45 | 110 | 55.8 | 0.4 | 11.4 | 14.0 | 24.1 |
| 65 | 2 | 30 | 240 | 60 | 0.4 | 45 | 110 | 54.2 | 0.4 | 11.8 | 8.3 | 13.8 |
| 66 | 2 | 30 | 240 | 60 | 0.4 | 45 | 110 | 53.0 | 0.4 | 10.9 | 10.8 | 17.9 |
| 67 | 2 | 30 | 240 | 60 | 0.4 | 45 | 110 | 49.7 | 0.4 | 10.9 | 10.7 | 18.2 |
| 68 | 2 | 30 | 240 | 60 | 0.4 | 45 | 110 | 49.6 | 0.4 | 11.2 | 12.0 | 21.3 |
| 69 | 2 | 30 | 240 | 60 | 0.4 | 45 | 110 | 50.9 | 0.4 | 11.3 | 12.6 | 21.6 |
| 70 | 2 | 30 | 240 | 60 | 0.4 | 45 | 110 | 53.5 | 0.4 | 11.5 | 8.1 | 13.5 |
| 71 | 2 | 30 | 200 | 60 | 0.4 | 45 | 110 | 43.5 | 0.5 | 10.7 | 7.2 | 11.2 |
| 72 | 2 | 30 | 200 | 60 | 0.4 | 45 | 110 | 51.2 | 0.4 | 11.3 | 8.0 | 13.0 |
| 73 | 2 | 30 | 200 | 60 | 0.4 | 45 | 110 | 48.0 | 0.5 | 11.5 | 8.1 | 12.9 |
| 74 | 2 | 30 | 200 | 60 | 0.4 | 45 | 110 | 53.1 | 0.5 | 11.2 | 8.9 | 13.5 |
| 75 | 2 | 30 | 200 | 60 | 0.4 | 45 | 110 | 51.1 | 0.4 | 10.8 | 8.5 | 13.9 |
| 76 | 2 | 30 | 200 | 60 | 0.4 | 45 | 110 | 48.8 | 0.4 | 10.9 | 7.9 | 12.7 |
| 77 | 3 | 30 | 230 | 60 | 0.145 | 45 | 110 | 38.3 | 0.6 | 12.9 | 7.2 | 15.3 |
| 78 | 3 | 30 | 230 | 60 | 0.145 | 45 | 110 | 39.2 | 0.6 | 12.2 | 6.2 | 12.7 |
| 79 | 3 | 30 | 230 | 60 | 0.145 | 45 | 110 | 38.1 | 0.6 | 12.8 | 7.7 | 15.7 |
| 80 | 3 | 30 | 230 | 60 | 0.145 | 45 | 110 | 37.1 | 0.6 | 12.7 | 5.8 | 12.5 |
| 81 | 3 | 30 | 230 | 60 | 0.145 | 45 | 110 | 37.1 | 0.6 | 12.7 | 5.4 | 12.2 |
| 82 | 3 | 30 | 230 | 60 | 0.145 | 45 | 110 | 39.6 | 0.6 | 13.3 | 5.4 | 12.8 |
| 83 | 3 | 30 | 230 | 60 | 0.315 | 45 | 110 | 36.8 | 0.6 | 12.8 | 8.7 | 16.2 |
| 84 | 3 | 30 | 230 | 60 | 0.315 | 45 | 110 | 41.2 | 0.6 | 12.8 | 11.9 | 22.4 |
| 85 | 3 | 30 | 230 | 60 | 0.315 | 45 | 110 | 39.9 | 0.6 | 12.7 | 8.5 | 16.1 |
| 86 | 3 | 30 | 230 | 60 | 0.315 | 45 | 110 | 39.8 | 0.6 | 13.0 | 8.2 | 15.4 |
| 87 | 3 | 30 | 230 | 60 | 0.315 | 45 | 110 | 41.1 | 0.6 | 13.2 | 8.0 | 15.0 |
| 88 | 3 | 30 | 230 | 60 | 0.315 | 45 | 110 | 39.4 | 0.6 | 13.5 | 7.3 | 14.5 |
| 89 | 3 | 30 | 210 | 60 | 0.06 | 45 | 110 | 49.4 | 0.5 | 13.6 | 5.0 | 15.8 |
| 90 | 3 | 30 | 210 | 60 | 0.06 | 45 | 110 | 32.9 | 0.6 | 12.5 | 4.1 | 13.8 |
| 91 | 3 | 30 | 210 | 60 | 0.06 | 45 | 110 | 38.7 | 0.6 | 14.5 | 5.7 | 17.5 |
| 92 | 3 | 30 | 210 | 60 | 0.06 | 45 | 110 | 41.1 | 0.6 | 12.6 | 4.2 | 13.7 |
| 93 | 3 | 30 | 210 | 60 | 0.06 | 45 | 110 | 44.2 | 0.6 | 14.3 | 4.6 | 16.1 |
| 94 | 3 | 30 | 210 | 60 | 0.06 | 45 | 110 | 46.4 | 0.6 | 14.7 | 5.6 | 17.8 |
| 95 | 3 | 30 | 210 | 60 | 0.06 | 45 | 110 | 44.2 | 0.6 | 12.2 | 2.8 | 11.7 |
| 96 | 3 | 30 | 210 | 60 | 0.06 | 45 | 110 | 46.5 | 0.5 | 12.9 | 7.8 | 21.1 |
| 97 | 3 | 30 | 210 | 60 | 0.06 | 45 | 110 | 43.5 | 0.5 | 12.9 | 8.4 | 23.4 |
| 98 | 3 | 30 | 210 | 60 | 0.06 | 45 | 110 | 38.9 | 0.6 | 12.2 | 4.2 | 13.3 |
| 99 | 3 | 30 | 210 | 60 | 0.06 | 45 | 110 | 29.2 | 0.4 | 7.8 | 6.2 | 16.1 |
| 100 | 3 | 30 | 210 | 60 | 0.06 | 45 | 110 | 27.6 | 0.5 | 8.8 | 6.1 | 16.3 |
| 101 | 3 | 30 | 230 | 60 | 0.06 | 45 | 110 | 46.1 | 0.4 | 13.0 | 7.3 | 17.4 |
| 102 | 3 | 30 | 230 | 60 | 0.06 | 45 | 110 | 46.3 | 0.5 | 14.7 | 9.5 | 23.4 |
| 103 | 3 | 30 | 230 | 60 | 0.06 | 45 | 110 | 49.1 | 0.4 | 13.4 | 8.0 | 19.1 |
| 104 | 3 | 30 | 230 | 60 | 0.06 | 45 | 110 | 46.6 | 0.5 | 12.5 | 7.9 | 17.7 |
| 105 | 3 | 30 | 230 | 60 | 0.06 | 45 | 110 | 45.8 | 0.5 | 12.7 | 8.9 | 20.5 |
| 106 | 3 | 30 | 230 | 60 | 0.06 | 45 | 110 | 46.9 | 0.5 | 13.5 | 9.4 | 22.3 |
| 107 | 3 | 30 | 230 | 60 | 0.23 | 90 | 110 | 63.3 | 0.4 | 14.4 | 8.7 | 18.5 |
| 108 | 3 | 30 | 230 | 60 | 0.23 | 90 | 110 | 60.5 | 0.4 | 14.5 | 10.4 | 22.3 |
| 109 | 3 | 30 | 230 | 60 | 0.23 | 90 | 110 | 57.1 | 0.5 | 13.8 | 9.4 | 18.9 |
| 110 | 3 | 30 | 230 | 60 | 0.23 | 90 | 110 | 45.1 | 0.3 | 6.4 | 9.9 | 15.7 |
| 111 | 3 | 30 | 230 | 60 | 0.23 | 90 | 110 | 53.8 | 0.5 | 13.2 | 11.2 | 23.0 |
| 112 | 3 | 30 | 230 | 60 | 0.23 | 90 | 110 | 58.6 | 0.4 | 13.1 | 11.7 | 24.1 |
| 113 | 3 | 30 | 210 | 60 | 0.23 | 90 | 110 | 50.8 | 0.5 | 13.4 | 11.1 | 23.1 |
| 114 | 3 | 30 | 210 | 60 | 0.23 | 90 | 110 | 49.1 | 0.5 | 13.8 | 8.3 | 18.0 |
| 115 | 3 | 30 | 210 | 60 | 0.23 | 90 | 110 | 54.8 | 0.5 | 14.6 | 9.4 | 20.3 |
| 116 | 3 | 30 | 210 | 60 | 0.23 | 90 | 110 | 45. | 0.4 | 10.8 | 8.4 | 15.1 |
| 117 | 3 | 30 | 210 | 60 | 0.23 | 90 | 110 | 52.1 | 0.4 | 12.9 | 9.2 | 19.1 |
| 118 | 3 | 30 | 210 | 60 | 0.23 | 90 | 110 | 54.7 | 0.5 | 16.4 | 10.0 | 23.7 |
| 119 | 3 | 30 | 210 | 60 | 0.315 | 45 | 110 | 44.9 | 0.5 | 13.9 | 7.2 | 15.2 |
| 120 | 3 | 30 | 210 | 60 | 0.315 | 45 | 110 | 47.4 | 0.4 | 12.5 | 6.3 | 11.4 |
| 121 | 3 | 30 | 210 | 60 | 0.315 | 45 | 110 | 48.4 | 0.5 | 13.9 | 10.6 | 22.8 |
| 122 | 3 | 30 | 210 | 60 | 0.315 | 45 | 110 | 47.5 | 0.4 | 12.4 | 7.1 | 13.0 |
| 123 | 3 | 30 | 210 | 60 | 0.315 | 45 | 110 | 44.4 | 0.5 | 12.5 | 7.1 | 13.3 |
| 124 | 3 | 30 | 210 | 60 | 0.315 | 45 | 110 | 51.1 | 0.5 | 14.3 | 11.6 | 23.9 |

**Table S2.** Sample count per print condition

| Condition | Iteration | T  (°C) | h  (mm) | Number of samples printed |
| --- | --- | --- | --- | --- |
| 1 | 1 | 200 | 0.10 | 9 |
| 2 | 1 | 200 | 0.15 | 1 |
| 3 | 1 | 220 | 0.06 | 6 |
| 4 | 1 | 220 | 0.10 | 1 |
| 5 | 1 | 220 | 0.20 | 1 |
| 6 | 1 | 220 | 0.15 | 13 |
| 7 | 1 | 220 | 0.30 | 7 |
| 8 | 1 | 220 | 0.40 | 1 |
| 9 | 1 | 220 | 0.25 | 1 |
| 10 | 1 | 240 | 0.10 | 6 |
| 11 | 2 | 200 | 0.40 | 6 |
| 12 | 2 | 200 | 0.315 | 6 |
| 13 | 2 | 240 | 0.23 | 6 |
| 14 | 2 | 240 | 0.40 | 6 |
| 15 | 2 | 240 | 0.315 | 6 |
| 16 | 3 | 210 | 0.06 | 12 |
| 17 | 3 | 210 | 0.23 | 6 |
| 18 | 3 | 210 | 0.315 | 6 |
| 19 | 3 | 230 | 0.06 | 6 |
| 20 | 3 | 230 | 0.23 | 6 |
| 21 | 3 | 230 | 0.315 | 6 |
| 22 | 3 | 230 | 0.145 | 6 |


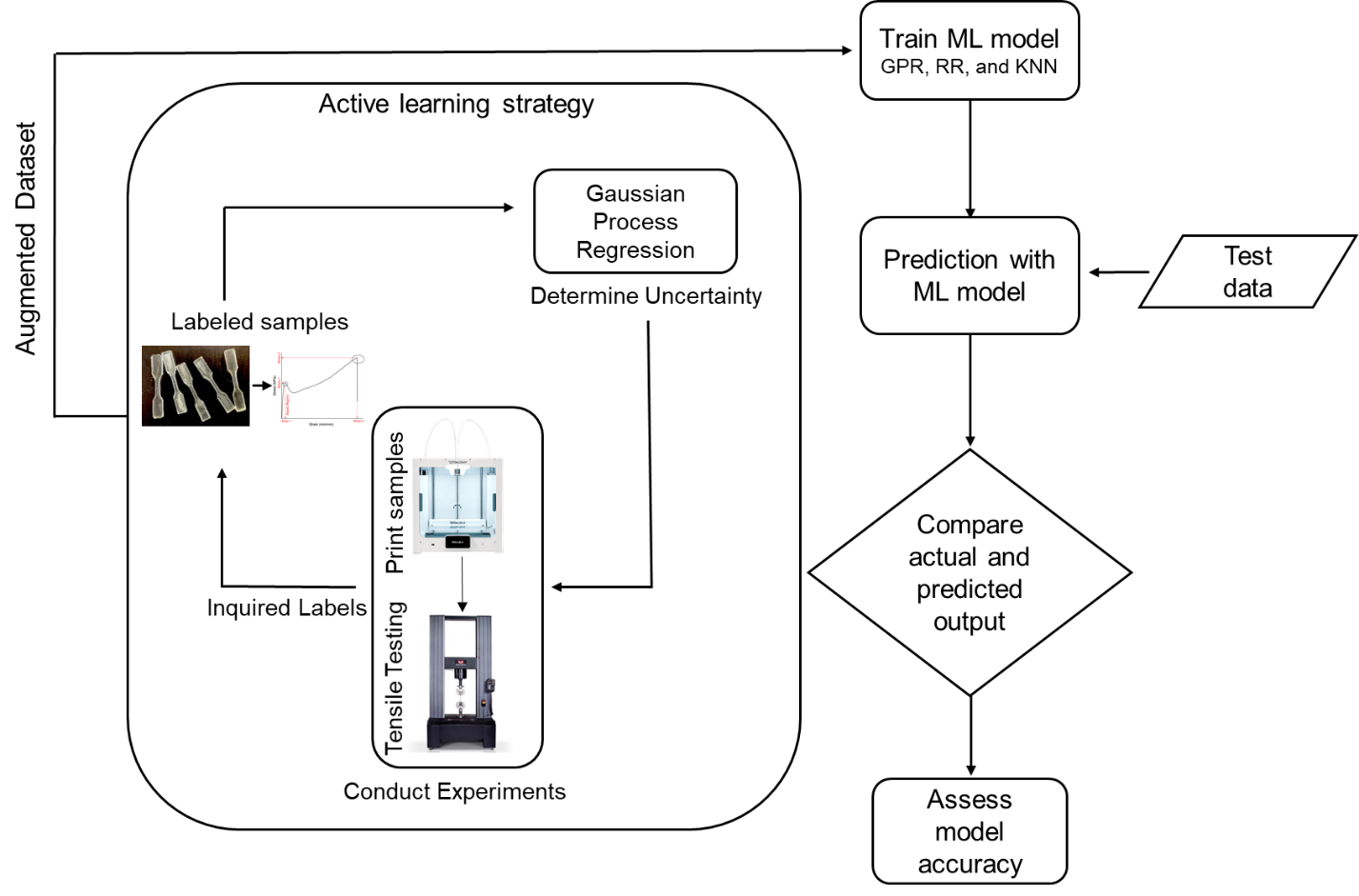


**Figure S2.** Process for training machine learning models using an active learning strategy.

**Section S1. Theory**

**S1.1 Review of regression algorithms**

The aim of a ML algorithm is to extract relationships between the input parameter space and the desired quantities of interest, or outputs. Let us consider a training dataset with $d$ input variables, vectorized as $x\in\mathcal{R}^{d}$ in the parameter space. The input-output pairs of the training dataset can be mathematically defined as $D_{\mathrm{train}}=\{\left( x_{1},y_{1} \right),\ldots,(x_{n},y_{n})\}$, where $n$ denotes the number of observations in the training dataset. ML algorithms try to learn a function,$f_{\theta}:x\mapsto y$, based on the observations in $D_{\mathrm{train}}$ that maps each input vector to the output space. Therefore, model-based ML methods solve the mathematical optimization problem described in Eq. 1.

$\theta^{*}\in\mathrm{argmin}_{\theta} \sum_{(x,y)\in D_{\mathrm{train}}} cost(y,f_{\theta}(x))$ (1)

For regression problems, the cost function in Eq. 1 is typically the sum of the squared differences between actual and predicted values. If $y$ is the experimental value and $f_{\theta}\left( x \right)$ is the predicted value

from the model, and assuming a linear input-output relationship $f_{\theta}\left( x \right)=\left\langle\theta,x \right\rangle$, the mathematical representation of the cost function is described in Eq. 2.

$\mathrm{cost}\left( y,f_{\theta}\left( x \right) \right)=\left( y-\left\langle\theta,x \right\rangle\right)^{2}$ (2)

The learned model $f_{\theta^{*}}$, where the optimal parameters $\theta^{*}$ result in the minimum value of the cost function, can make predictions for testing samples (i.e., conditions that are not part of the training dataset).

**S1.1.1 Linear regression (LR)**

LR finds the linear relationship between the independent and dependent variables. It should be noted that LR minimizes the squared error and may not always provide the best representation of the data with a line. Two independent variables required the use of multiple LR in this work. Eq. 3 describes the mathematical representation of multiple LR for $p$ independent variables, where $f_{\theta}\left( x_{i} \right)$ represents the linear relationship between the input and output variables^1^.

$f_{\theta}\left( x_{i} \right)= \beta_{o}+ \beta_{1}x_{1i}+ \beta_{2}x_{2i}+\ldots+ \beta_{p}x_{\mathrm{pi}}+e_{i}$ (3)

Here, $\beta_{o}$ is a constant and predicted value when all independent variables are equal to zero. Each independent variable has a unique coefficient, $\beta$. $e_{i}$ represents the residual or error term, which is the difference between the actual and the predicted value.

**S1.1.2. Ridge regression (RR)**

RR, a model-based regression method, minimizes the cost function similarly to least squares method. However, RR balances model complexity and model accuracy by employing a regularization term that measures the norm of the parameter vector $\theta$ (i.e., $\left\| \theta\right\|_{2}^{2}=\theta_{1}^{2}+\ldots+\theta_{d}^{2}$). Therefore, the resulting optimization problem for RR minimizes the cost function in Eq. 4.

$\sum_{(x,y)\in D_{\mathrm{train}}} \left( y-\left\langle\theta,x \right\rangle\right)^{2}+\lambda\left\| \theta\right\|_{2}^{2}$ (4)

Here, $\lambda\geq0$ is a tuning parameter, or hyperparameter, that is determined separately. Like the least squares method, RR tries to find the coefficient estimates that fit the data by reducing $\left( y-\left\langle\theta,x \right\rangle\right)^{2}$. The second term, $\lambda\left\| \theta\right\|_{2}^{2}$, is called a shrinkage penalty. When $\lambda=0$, the penalty term does not have any effect and RR will produce the least squares estimates. On the other hand, when $\lambda$ is relatively large, the impact of shrinkage penalty grows and the RR coefficient estimates approach zero, which means the regression model does not learn any useful pattern from the training dataset. Therefore, it is critical to select an appropriate value for $\lambda$. Cross-validation provides a simple way to find the tuning parameter. A grid of $\lambda$ values is chosen, and the cross-validation error is determined for each value of $\lambda$. The tuning parameter value that has the smallest cross-validation error is selected^2^.

**S1.1.3. K-nearest neighbor (KNN)**

Unlike RR, some ML methods are instance-based rather than being model-based. KNN is one such algorithm, which does not require definition of the cost functions, thus eliminating an optimization task. For new test data points, KNN looks for the closest points in the training dataset. The algorithm then sorts the closest training data points by calculating the distance between them and the new test data point. Various approaches are available to calculate the distance, but the simplest and the most popular is the Euclidean distance. The algorithm then calculates the average of outputs for the $k$ nearest training samples^3^. Let us take $x_{q}$ as a test data point or sample. The KNN algorithm finds the $k$ closest points to the test point, denoted by $x_{1},\ldots,x_{k}$, to predict the output value as described in Eq. 5.

$f\left( x_{q} \right)= \frac{\sum_{i=1}^{k} y_{i}}{k}$ (5)

A refinement to KNN is to weight the contributions of each of the neighbors based on their distance from the query point $x_{q}$, with closer neighbors receiving greater weight^4^. The contribution of each neighbor may be determined according to the inverse square of its distance from $x_{q}$. For the weighted version of KNN, Eq. 5 is revised to Eq. 6.

$f\left( x_{q} \right)= \frac{\sum_{i=1}^{k} w_{i}y_{i}}{\sum_{i=1}^{k} w_{i}}$ (6)

where,

$w_{i}= \frac{1}{\left\| x_{q}- x_{i} \right\|^{2}}$ (7)

The denominator in Eq. 6 is a constant that normalizes the contributions of the various weights. It is possible for all $k$ training samples to influence $f \left( x_{q} \right)$, although distant training samples will have minimal effect, and closer samples will have a greater effect.

**S1.1.4. Gaussian process regression (GPR)**

GPR is a probabilistic ML model that, unlike RR and KNN, can provide uncertainty measures over predictions. The Gaussian process is a distribution over a function that is defined by mean and covariance functions. The mean function, $m(x)$, represents the average of all the functions in the distribution determined at input $x$. The covariance function, $\kappa(x,x^{'})$, reflects the similarity between any two input points $x$ and $x'$. The function $\kappa$ is generally known as the kernel of the Gaussian process^5^. The choice of an appropriate kernel function is based on the smoothness and expected pattern in the dataset. A widely used assumption is that the correlation between two points decays with the distance between two points, meaning that closer points behave more similarly than points that reside further away from each other. The radial basis function (RBF) kernel is a common choice fulfilling this assumption and is defined as

$\kappa\left( x, x' \right)= \sigma_{f}^{2}exp(-\frac{\left\| x-x' \right\|^{2}}{2\lambda^{2}})$ (8)

The hyperparameters $\lambda$ (length-scale) and $\sigma_{f}$ (signal variance) can be varied to increase or decrease the correlations between the data points, which in turn change the resulting function.

GPR assumes that the mapping function $f(x)$ is distributed as a Gaussian process according to Eq. 9.

$f\left( x \right)=GP (m\left( x \right), \kappa\left( x,x^{'} \right))$ (9)

With the training dataset $D_{\mathrm{train}}=\left\{ \left( x_{1}, y_{1} \right),\ldots,(x_{n}, y_{n}) \right\}$, predictions at a test data point $x$ can be obtained using the following equation.

$p\left( \hat{y} | x,D_{\mathrm{train}} \right)=N(\mu, \sigma^{2})$ (10)

In Eq. 10, the mean of the Gaussian distribution satisfies $\mu=\alpha^{T}K^{-1}y$, where $K$ is the covariance matrix between all the observed points in $D_{\mathrm{train}}$, $\alpha$ is a vector that contains values of the kernel function between $x$ and the observed points, and $y$consists of all the outputs in $D_{\mathrm{train}}$. The variance of this distribution can also be calculated using the vector $\alpha$ and the matrix $K$.

**S1.2 Active learning (AL) with GPR**

AL enables ML algorithms to choose the data they learn from to perform better with fewer training samples^6^. In this work, we used pool-based AL^7^. The process starts with taking a small number of labeled training data points to train a surrogate model using GPR. Samples from the design space are selected for analyzing the predicted uncertainties. Since the surrogate model is trained using GPR, it is capable of predicting the uncertainty along with the means of the outputs. According to the exploration strategy, the data points in the design space that have the largest uncertainty should be taken for the next experiments^8^. The results from the experiments are used as the new training data points in the surrogate model. The model continues to iterate until an augmented training dataset is obtained for training high-quality ML models.

**Figure S3.** Stress-strain curves of the samples printed at test conditions A, B, C, D, and E.





**Figure S4.** Heatmap showing the correlation between the input and output parameters in test dataset.


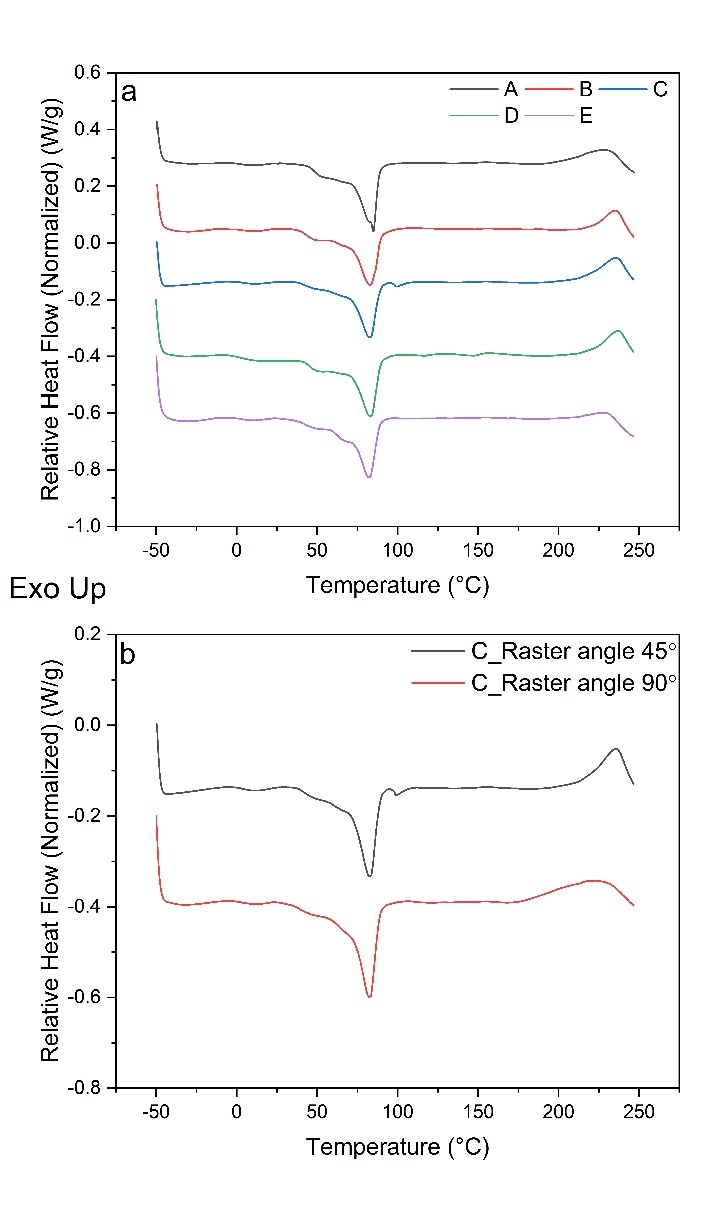


**Figure S5.** Representative heating curves of a. Test samples print at Condition A, B, C, D, and E; b. Comparison of heating curves of Condition C samples printed at raster angles 45° and 90°.





**Figure S6.** Assessment of model accuracy by comparing actual and predicted output values of the test samples from LR, RR, GPR, and KNN.

**References**

1. Tranmer, M., Murphy Jen, Elliot, M. & Pampaka, M. *Multiple Linear Regression*. (Cathie Marsh Institute Working Paper 2020-01, 2020).

2. de Vlaming, R. & Groenen, P. J. F. The Current and Future Use of Ridge Regression for Prediction in Quantitative Genetics. *Biomed Res Int* **2015**, 143712 (2015).

3. Kramer, O. K-Nearest Neighbors. in *Dimensionality Reduction with Unsupervised Nearest Neighbors* (ed. Kramer, O.) 13–23 (Springer Berlin Heidelberg, 2013). doi:10.1007/978-3-642-38652-7_2.

4. Hechenbichler, K. & Schliep, K. *Weighted k-Nearest-Neighbor Techniques and Ordinal Classification*. vol. 399 http://nbn-resolving.de/urn/resolver.pl?urn=nbn:de:bvb:19-epub-1769-9 (2004).

5. Jäkel, F., Schölkopf, B. & Wichmann, F. A. A tutorial on kernel methods for categorization. *J Math Psychol* **51**, 343–358 (2007).

6. Settles, B. Active Learning Literature Survey. *Computer Sciences Technical Report* **1648**, (2009).

7. Settles, B. Active Learning. *Synthesis Lectures on Artificial Intelligence and Machine Learning* **6**, 1–114 (2012).

8. Lookman, T., Balachandran, P. v, Xue, D. & Yuan, R. Active learning in materials science with emphasis on adaptive sampling using uncertainties for targeted design. *NPJ Comput Mater* **5**, 21 (2019).
